# IL-7Rα on CD4^+^ T cells is required for their survival and the pathogenesis of experimental autoimmune encephalomyelitis

**DOI:** 10.1186/s12974-024-03224-2

**Published:** 2024-10-08

**Authors:** Gholamreza Azizi, Bram Van den Broek, Larissa Lumi Watanabe Ishikawa, Hamed Naziri, Reza Yazdani, Guang-Xian Zhang, Bogoljub Ciric, Abdolmohamad Rostami

**Affiliations:** Department of Neurology, Thomas Jefferson University, 900 Walnut Street, Suite 300, Philadelphia, PA 19107 USA

**Keywords:** IL-7 receptor alpha, Experimental autoimmune encephalomyelitis, Multiple sclerosis, CD4^+^ T cell

## Abstract

**Background:**

The IL-7 receptor alpha (IL-7Rα) binds both IL-7 and thymic stromal lymphopoietin (TSLP). IL-7Rα is essential for the development and survival of naive CD4^+^ T cells and their differentiation to effector/memory CD4^+^ T cells. Mice lacking IL-7Rα have severe lymphopenia and are resistant to experimental autoimmune encephalomyelitis (EAE), a model for multiple sclerosis. However, it has been reported that IL-7Rα on peripheral CD4^+^ T cells is disposable for their maintenance and EAE pathogenesis, which does not align with the body of knowledge on the role of IL-7Rα in the biology of CD4^+^ T cells. Given that a definitive study on this important topic is lacking, we revisited it using a novel approach, an inducible knockout of the IL-7Rα gene in CD4^+^ T cells.

**Methods:**

We generated *Il7ra*^fl/fl^/CD4CreER^T2^ double transgenic mouse line (henceforth CD4^Δ*Il7ra*^), susceptible to tamoxifen-induced knockout of the IL-7Rα gene in CD4^+^ T cells. CD4^Δ*Il7ra*^ mice were immunized with MOG_35 − 55_ for EAE induction and monitored for disease development. The expression of IL-7Rα, CD4^+^ T cell numbers, and MOG_35 − 55_-specific CD4^+^ T cell response was evaluated in the central nervous system (CNS) and lymphoid tissues by flow cytometry. Additionally, splenocytes of CD4^Δ*Il7ra*^ mice were stimulated with MOG_35 − 55_ to assess their proliferative response and cytokine production by T helper cells.

**Results:**

Loss of IL-7Rα from the surface of CD4^+^ T cells in CD4^Δ*Il7ra*^ mice was virtually complete several days after tamoxifen treatment. The loss of IL-7Rα in CD4^+^ T cells led to a gradual and substantial decrease in their numbers in both non-immunized and immunized CD4^Δ*Il7ra*^ mice, followed by slow repopulation up to the initial numbers. CD4^Δ*Il7ra*^ mice did not develop EAE. We found a decrease in the total numbers of TNF-, IFN-γ-, IL-17 A-, and GM-CSF-producing CD4^+^ T cells and regulatory T cells in the spleens and CNS of immunized CD4^Δ*Il7ra*^ mice. Tracking MOG_35 − 55_-specific CD4^+^ T cells revealed a significant reduction in their numbers in CD4^Δ*Il7ra*^ mice and decreased proliferation and cytokine production in response to MOG_35 − 55_.

**Conclusion:**

Our study demonstrates that IL-7Rα on peripheral CD4^+^ T cells is essential for their maintenance, immune response, and EAE pathogenesis.

**Supplementary Information:**

The online version contains supplementary material available at 10.1186/s12974-024-03224-2.

## Introduction

The IL-7 receptor (IL-7R) complex comprises the high-affinity IL-7Rα chain (CD127) and the common cytokine gamma-chain (γc; CD132). IL-7Rα is also part of the receptor for thymic stromal lymphopoietin (TSLP), which supports the growth of T- and B-cell precursors [1, 2]. IL-7/IL-7R signaling is critical for T and B cell development [3–5]. IL-7/IL-7R signaling promotes T cell survival by inhibiting the mitochondrial apoptotic pathway *via* upregulation of expression of the anti-apoptotic Bcl-2 family molecules and downregulation of expression of pro-apoptotic molecules [6–8]. Consistent with the essential role of IL-7/IL-7R signaling in lymphopoiesis, mice lacking IL-7Rα exhibit profound lymphopenia [9, 10].

IL-7R signaling promotes the expansion and survival of developing CD4^+^ T cells in the thymus [7, 11]. In the periphery, IL-7R signaling supports the survival of naive CD4^+^ T cells and their differentiation into effector cells [12]. Finally, IL-7/IL-7R signaling is needed for maintenance of long-lived memory CD4^+^ T cells [13, 14]. IL-7Rα deletion resulted in delayed spleen and lymph node (LN) T cell proliferation following stimulation [15], while IL-7/IL-7R signaling promoted IFN-γ and GM-CSF secretion by CD4^+^ T cells in vitro [16]. An exposure of CD4^+^ T cells to IL-7 led to loss of IL-7Rα from their surface, but the receptor was re-expressed after several days [17].

Research has demonstrated that the IL-7/IL-7Rα pathway plays a role in multiple sclerosis (MS) and its animal model, experimental autoimmune encephalomyelitis (EAE) [16, 18, 19]. In humans, certain polymorphisms in the IL-7Rα gene have been associated with an increased risk of developing MS [20]. High IL-7 expression was detected in the CNS during EAE, with IL-7 driving the T helper (Th) 17 to Th1 cell plasticity [16]. Targeting IL-7/IL-7Rα was beneficial in EAE with reduced numbers of peripheral naïve and activated T cells [21]. However, IL-7/anti-IL-7 mAb complexes selectively expanded Th1 cells without affecting Th17 cells or the development of EAE [16, 22]. It has been reported that IL-7Rα on peripheral CD4^+^ T cells is disposable for their maintenance and EAE pathogenesis and that IL-7Rα on either hematopoietic (e.g., T cells) or non-hematopoietic cells (e.g., oligodendrocytes and astrocytes) is sufficient for severe EAE to develop [19]. These findings are not necessarily in agreement with the body of knowledge on the role of IL-7Rα in the biology of CD4^+^ T cells, and a definitive study on this important topic is lacking.

Here, we revisited this matter using a novel experimental approach, an inducible knockout of the IL-7Rα gene in peripheral CD4^+^ T cells. We investigated the role of IL-7Rα in maintaining CD4^+^ T cells in the periphery and tested how conditional knockout of the IL-7Rα gene in peripheral CD4^+^ T cells impacts an immune response using the EAE model. In contrast to the previous report [19], we show that loss of IL-7Rα in CD4^+^ T cells causes a gradual and long-lasting reduction in their numbers, diminished myelin-specific CD4^+^ T cell response and resistance to EAE development.

## Materials and methods

### Mice

C57BL/6 (B6), *Il7ra*^fl^ [23] and CD4CreER^T2^ [22] mouse lines were purchased from the Jackson Laboratory. *Il7ra*^fl/fl^/CD4CreER^T2^ double transgenic line (henceforth designated CD4^Δ*Il7ra*^) was generated in-house. Both male and female mice at 2–5 months of age were used in experiments; CD4^Δ*Il7ra*^ and control mice were matched for age and sex in each experiment. Experiments were performed with prior Institutional Animal Care and Use Committee approval.

### Tamoxifen preparation and treatment

Tamoxifen (1 g; Sigma-Aldrich) was dissolved in ethanol (1 mL) and then added to 9 mL of corn oil. The solution was heated at 55 °C for 30 min until tamoxifen was dissolved and then diluted with 10 mL of corn oil to a final tamoxifen concentration of 50 mg/mL. As specified in the figure legends, Tamoxifen solution was administered to mice by oral gavage.

### EAE induction

EAE was induced in 10-12-week-old CD4^Δ*Il7ra*^ and CD4CreER^T2^ (control) mice by subcutaneous injection of 200 µg of MOG_35 − 55_ (GenScript, CA, USA) in Complete Freund’s Adjuvant (Thermo Scientific) containing heat-inactivated *Mycobacterium tuberculosis* H37Ra (5 mg/ml, Difco Lab). Mice received 200 ng of pertussis toxin (Sigma-Aldrich) intraperitoneally (i.p.) on days 0 and 2 postimmunization (d.p.i.), and their clinical signs were assessed daily according to the following scoring scale: 0, no sign of clinical disease; 1, paralysis of the tail; 2, paralysis of one hindlimb; 3, paralysis of both hindlimbs; 4, paralysis of the abdomen; 5, death.

### Isolation of CNS mononuclear cells

Mice were perfused through the left ventricle with cold PBS, and brains and spinal cords were collected and digested in Liberase (Sigma-Aldrich) solution for 30 min at 37 °C. Cells were suspended in a one-layer 40% Percoll (GE Healthcare, Little Chalfont, U.K.) and spun at 500 × *g* for 30 min at 24 °C [24].

### Flow cytometry and intracellular staining

Cells were activated with PMA (50 ng/ml; Sigma-Aldrich), Ionomycin (500 ng/ml; Sigma-Aldrich), and GolgiPlug (1 µg/ml; BD Biosciences) at 37 °C for 5 h. Cells were then washed, stained with antibodies (Abs) for surface markers (Table S1), and, after fixation/permeabilization with the FIX & PERM™ Cell Permeabilization Kit (Invitrogen) or the Foxp3 Transcription Factor Staining Buffer Set (Invitrogen), stained with Abs for intracellular antigens (Table S1). Data were acquired on a FACSAria Fusion (BD Biosciences) and analyzed by FlowJo software (TreeStar).

### Proliferation assay

Splenocytes were seeded in a flat-bottom 96-well plate (250 × 10^3^ cells per well) in IMDM medium containing 10% heat-inactivated fetal calf serum, 2 mM L-glutamine, 100 U/mL penicillin, 100 µg/mL streptomycin, and 50 µM β-mercaptoethanol. Splenocyte proliferation was induced either with MOG_35-55_ (25 µg/ml) or anti-CD3 and anti-CD28 monoclonal Abs (mAbs) (0.2 µg/mL) as a positive control. Relative cell proliferation was measured by CyQUANT™ XTT Cell Viability Assay (Invitrogen™) according to the manufacturer’s instructions.

### Cytokine quantification

Splenocytes were cultured and stimulated as mentioned in the proliferation assay section. After 4 days, cell culture supernatants were collected; and TNF, IFN-γ, IL-17, IL-10, and GM-CSF concentrations were measured by ELISA kits (R&D Systems) according to the manufacturer’s instructions.

### MHC tetramer staining

Splenocytes and LN cells were harvested at the preclinical phase (8 d.p.i.), clinical onset (12 d.p.i.), and peak (18 d.p.i.) of EAE; CNS cells were harvested at the onset and peak of EAE in control mice. 1 × 10^6^ cells were stained with I-Ab MOG_35-55_ MHC tetramer-PE (MBL, Japan) along with Abs against CD4 and CD45 and analyzed by flow cytometry.

### Statistical analysis

Statistical analysis was conducted using GraphPad Prism 8 software. Data were analyzed using an unpaired, two-tailed Student’s t-test between two groups and a Kruskal-Wallis test between three or more groups for clinical score analysis. *P* ≤ 0.05 was considered significant. Data represent means ± SEM.

## Results

### An efficient and specific knockout of IL-7Rα in CD4+ T cells

To knockout the IL-7Rα gene in CD4^+^ T cells, we crossed *Il7ra*^fl^ and CD4CreER^T2^ mouse lines and generated the CD4^Δ*Il7ra*^ double transgenic line, which is susceptible to tamoxifen-induced knockout of *Il7ra* in cells expressing CD4.

**Fig. 1:**
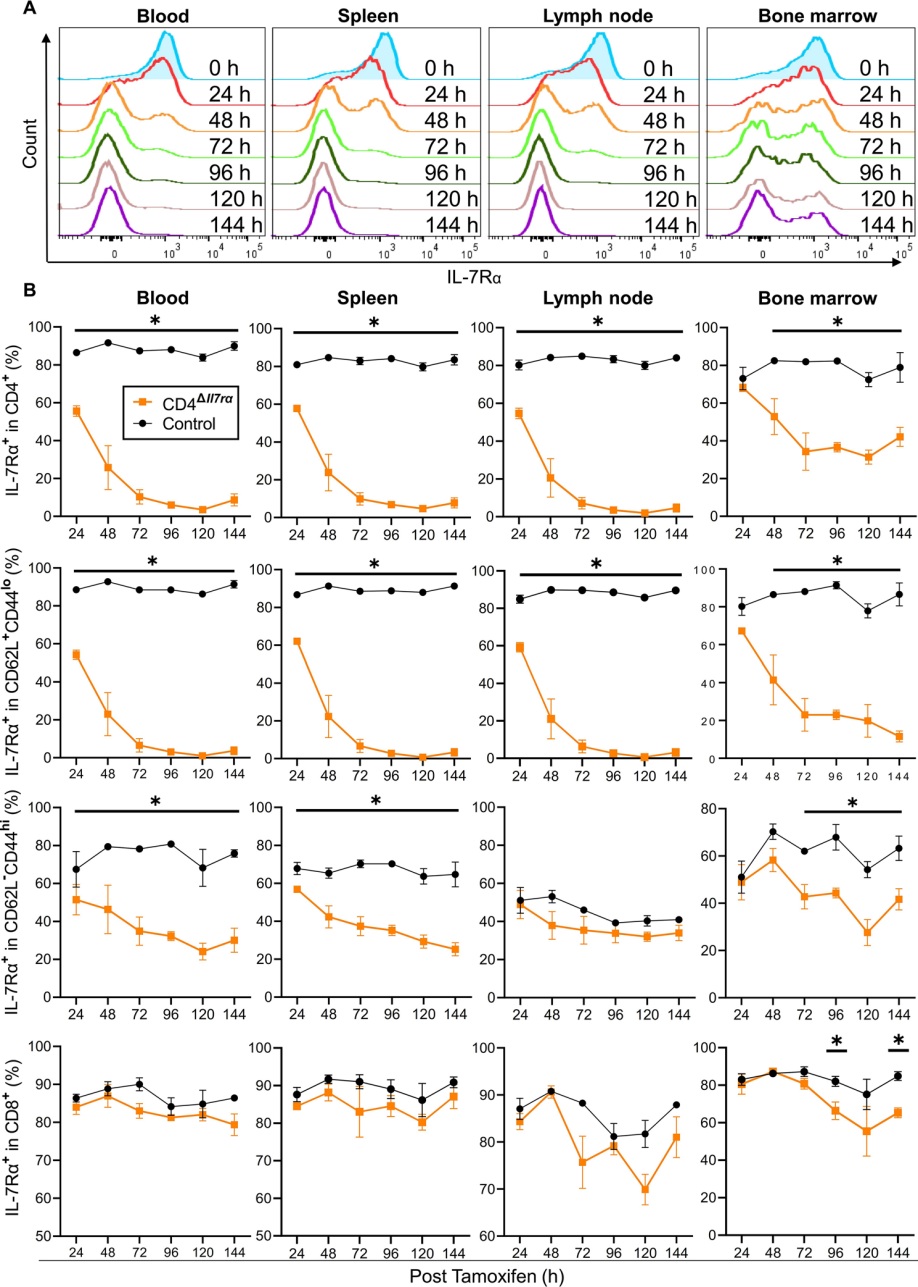
Tamoxifen-induced loss of IL-7Rα on CD4^+^ T cells in CD4^Δ*Il7ra*^ mice. Adult (3–5 months old) male and female CD4^Δ*Il7ra*^ and control CD4CreER^T2^ mice were treated once with 250 mg/kg of tamoxifen *via* oral gavage. Test and control mice sacrificed at each time point were matched by sex and age. Mice were sacrificed every 24 h for 6 days and evaluated by flow cytometry for IL-7Rα on total, naïve (CD62L^+^CD44^lo^), and effector/memory (CD62L^−^CD44^hi^) CD4^+^ T cells and total CD8^+^ T cells from the blood, spleen, LNs, and BM. (**A**) Flow cytometry histograms showing IL-7Rα staining on total CD4^+^ T cells after tamoxifen treatment. Data for a single representative mouse at each time point is shown. (**B**) Proportions (3–4 mice/group/time point) of IL-7Rα^+^CD4^+^ T cells and total CD8^+^ T cells. Data are presented as mean ± SEM. The unpaired t-test was used for sample comparisons. *p ≤ 0.05

To characterize the knockout of the IL-7Rα gene, an adult (2–5 months old) CD4^Δ*Il7ra*^ and CD4CreER^T2^ mice were treated once with tamoxifen by oral gavage and sacrificed every 24 h for 6 days. IL-7Rα presence was assessed by flow cytometry on total, naïve (CD62L^+^CD44^lo^), and effector/memory (CD62L^−^CD44^hi^) CD4^+^ cells and total CD8^+^ T cells from the blood, spleen, LN, and bone marrow (BM). IL-7Rα staining on total and naïve CD4^+^ T cells of CD4^Δ*Il7ra*^ mice was already decreased at 24 h after tamoxifen treatment and fully waned by 120 h in the blood, spleen, and LNs (Fig. 1A). The loss of IL-7Rα on total BM CD4^+^ T cells was less efficient than in the periphery, but naïve BM CD4^+^ T cells became IL-7Rα^−^, as did those in the periphery (Fig. 1B). Unexpectedly, a portion (~ 25%) of effector/memory CD4^+^ T cells in the blood, spleen, and BM remained IL-7Rα^+^. Fewer effector/memory CD4^+^ T cells in LNs were IL-7Rα^+^ (40–50%) than in other organs (70–80%) before tamoxifen treatment, and after the treatment, they remained IL-7Rα^+^ during the 6-day follow-up. Although the loss of IL-7Rα on effector/memory CD4^+^ T cells was incomplete, the loss on total CD4^+^ T cells was virtually complete because the effector/memory cells constituted only a minor portion (typically < 10%) of total CD4^+^ T cells (Fig. S1 and Fig. S2A). Except for a slight reduction in BM, the numbers of IL-7Rα^+^ CD8^+^ T cells remained unaffected, confirming that the knockout was limited to CD4^+^ T cells (Fig. 1B).

Given that IL-7Rα signaling contributes to the survival and proliferation of T cells, we determined whether the IL-7Rα gene knockout affected the numbers of CD4^+^ T cells. We compared frequencies of total, naïve, effector/memory, Treg CD4^+^, and total CD8^+^ T cells of CD4^Δ*Il7ra*^ and control mice. Six days post tamoxifen treatment, CD4^+^ T cell frequencies among mononuclear cells from the blood, spleen, LN, and BM of CD4^Δ*Il7ra*^ mice were not significantly reduced. The reduction was significant in BM cells, but the numbers of CD4^+^ T cells among BM cells were small (~ 1%), making this reduction likely inconsequential (Fig. S1).

Our data show that even a single tamoxifen treatment of CD4^Δ*Il7ra*^ mice resulted in a loss of IL-7Rα on CD4^+^ T cells after 4–5 days. This led to a trend toward reduced numbers of CD4^+^ T cells, but 6 days after the tamoxifen treatment, the reduction was not yet significant (Fig. S1). Our data from longer observations show that the loss of CD4^+^ T cells eventually becomes pronounced (Fig. S2B). We observed a substantial decrease (45–60%) in the proportion of CD4^+^ T cells among mononuclear cells within one month following tamoxifen treatment. In CD4^Δ*Il7ra*^ mice, both naïve and those immunized for EAE induction, the proportions of IL-7Rα^+^ CD4^+^ T cells returned to almost normal levels about two months after tamoxifen treatment (Fig. S3A and B).

### IL-7Rα knockout in CD4+ T cells precludes EAE

**Fig. 2:**
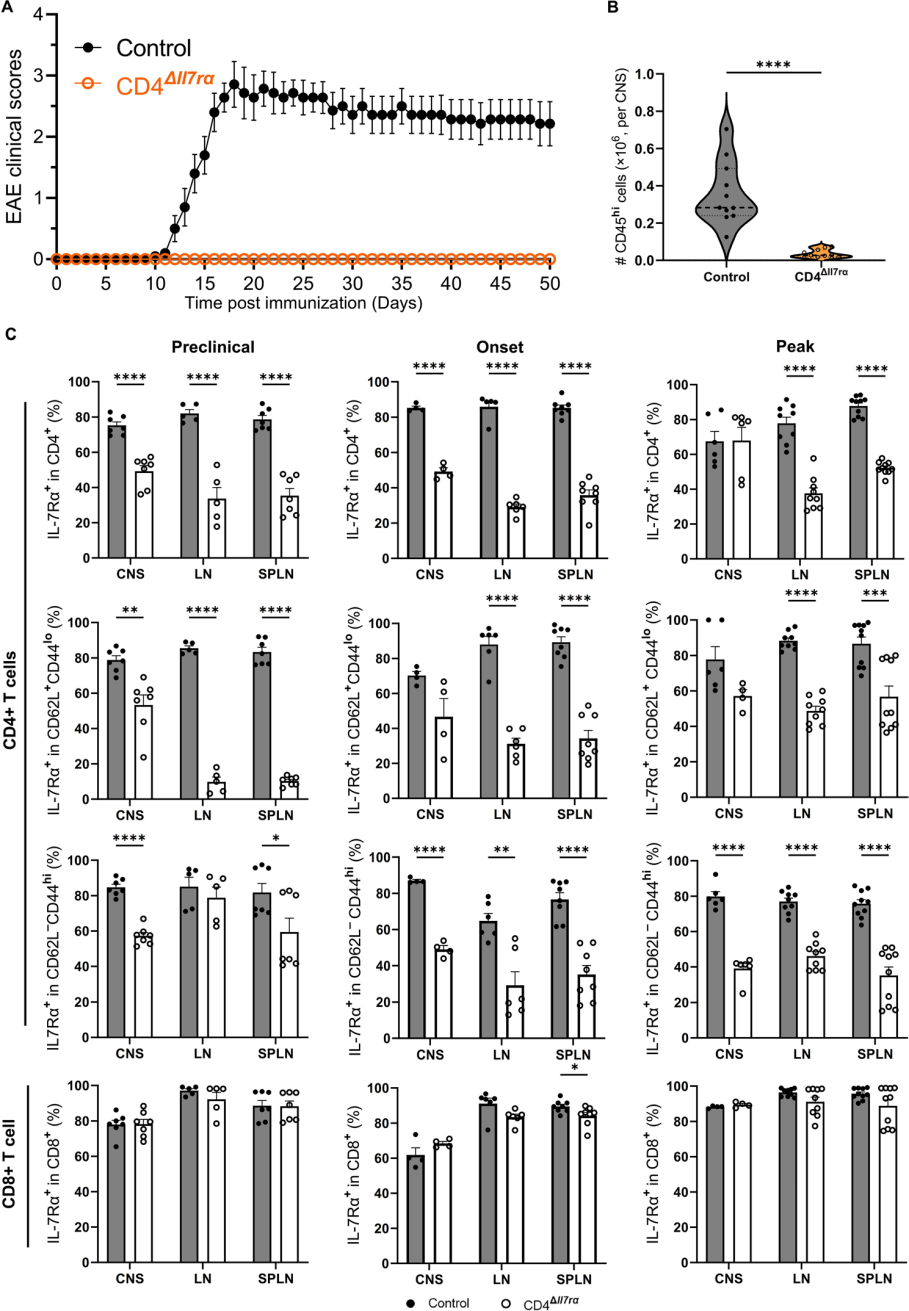
IL-7Rα gene knockout in CD4^+^ T cells abrogates EAE. Mice were treated with two doses of tamoxifen for two consecutive days, and IL-7Rα loss on blood CD4^+^ T cell was confirmed 5 days later. Mice were then immunized for EAE induction. (**A**) EAE clinical course in tamoxifen-pretreated CD4^Δ*Il7ra*^ and CD4CreER^T2^ control mice. (**B**) Numbers of CD45^hi^ cells isolated from the CNS at EAE peak in control mice. (**C**) The expression of IL-7Rα on CD4^+^ and CD8^+^ T cells from the spleen, LNs, and CNS at the preclinical phase (8 d.p.i.), clinical onset (12 d.p.i.), and EAE peak (18 d.p.i.) in control mice. Results are expressed as the mean ± SEM with *n* ≥ 5 per group from 3 independent experiments. Data were analyzed by Student’s t-test; *P < 0.05; **P < 0.01; ***P < 0.001; ****P < 0.0001

IL-7Rα signaling is necessary for normal T cell development, survival, and function; as a result, conventional whole-body IL-7Rα gene knockout mice have a severely deficient immune system [9, 25] that does not support EAE pathogenesis [19, 26]. However, a study has reported that IL-7Rα expression by either hematopoietic or non-hematopoietic cells was sufficient for typical EAE to develop [19]. Further, according to this report, mice lacking IL-7Rα on CD4^+^ T cells in the periphery developed typical EAE. To validate this finding, we used our CD4^Δ*Il7ra*^ mice, which have normal immune systems, including T cell compartment. CD4^+^ T cell-specific knockout of the IL-7Rα gene was achieved by tamoxifen treatment several days before immunization for EAE induction. Virtually all naïve CD4^+^ T cells lost IL-7Rα several days after tamoxifen treatment, while only a minority of effector/memory CD4^+^ T cells remained IL-7Rα^+^ (Fig. 1). Notably, numbers of CD4^+^ T cells after tamoxifen treatment gradually declined, but at the time of immunization (5 days post-treatment), their numbers were still similar to those in control mice. CD4^Δ*Il7ra*^ mice did not develop EAE (Fig. 2A and Fig. S3C), and at disease peak in control mice, only a small number of CD45^hi^ immune cells was infiltrated in the CNS of CD4^Δ*Il7ra*^ mice (Fig. 2B).

Given that IL-7Rα expression on CD4^+^ T cells, as a population, can be renewed either by proliferation of cells that did not lose it after tamoxifen treatment (e.g., some effector/memory cells) or by *de novo* development of naïve CD4^+^ T cells from BM precursors, we observed the numbers of IL-7Rα^+^CD4^+^ T cells in CD4^Δ*Il7ra*^ mice after immunization. There was a gradual increase in the proportion of IL-7Rα^+^CD4^+^ T cells in the blood; at the time of onset of clinical disease in control mice, approximately 20% of CD4^+^ T cells were IL-7Rα^+^, but that number approached the normal value (70% in CD4^Δ*Il7ra*^ mice vs. 90% in control mice) by day 50 p.i. (Fig. S3B). In CD4^Δ*Il7ra*^ mice, proportions of IL-7Rα^+^CD4^+^ T cells from the spleen, LNs, and CNS at the preclinical phase, clinical onset, and the peak of EAE (in control mice) were notably reduced (Fig. 2C). When we analyzed CD4^+^ Th cells stratified by cytokine production, we found substantial proportions of IL-7Rα^−^ cells that produce cytokines (Fig. S4). This suggests that IL-7Rα^−^CD4^+^ T cells retained the capacity to secrete TNF, IFN-γ, IL-17, and GM-CSF, at least after their stimulation with PMA and Ionomycin. Notably, the proportion of IL-7Rα^+^ effector/memory CD4^+^ T cells in LNs did not decrease during the preclinical phase of EAE (8 d.p.i. /12 days after second tamoxifen treatment) compared to control mice. This is similar to naïve mice treated with one dose of tamoxifen and analyzed 6 days later. This similarity suggests that the resistance of effector/memory cells to knockout of the IL-7Rα gene is not due to insufficient tamoxifen treatment or a short period between the treatment and analyses. At later time points, 12 d.p.i. and 18 d.p.i. proportions of IL-7Rα^+^ effector/memory cells in LNs were decreased, similar to the decrease in the spleen (Fig. 2C).

These data show that tamoxifen-pretreated and immunized CD4^Δ*Il7ra*^ mice experience a loss of IL-7Rα in CD4^+^ T cells similar to those not immunized. The loss of IL-7Rα in CD4^+^ T cells abrogates EAE development, demonstrating that, at least in this CD4^+^ T cell-driven model, IL-7Rα expression by CD4^+^ T cells is essential for disease to develop and that IL-7Rα expressed by other cell types could not mediate EAE pathogenesis.

### IL-7Rα gene knockout in CD4+ T cells leads to a reduction in their numbers

**Fig. 3:**
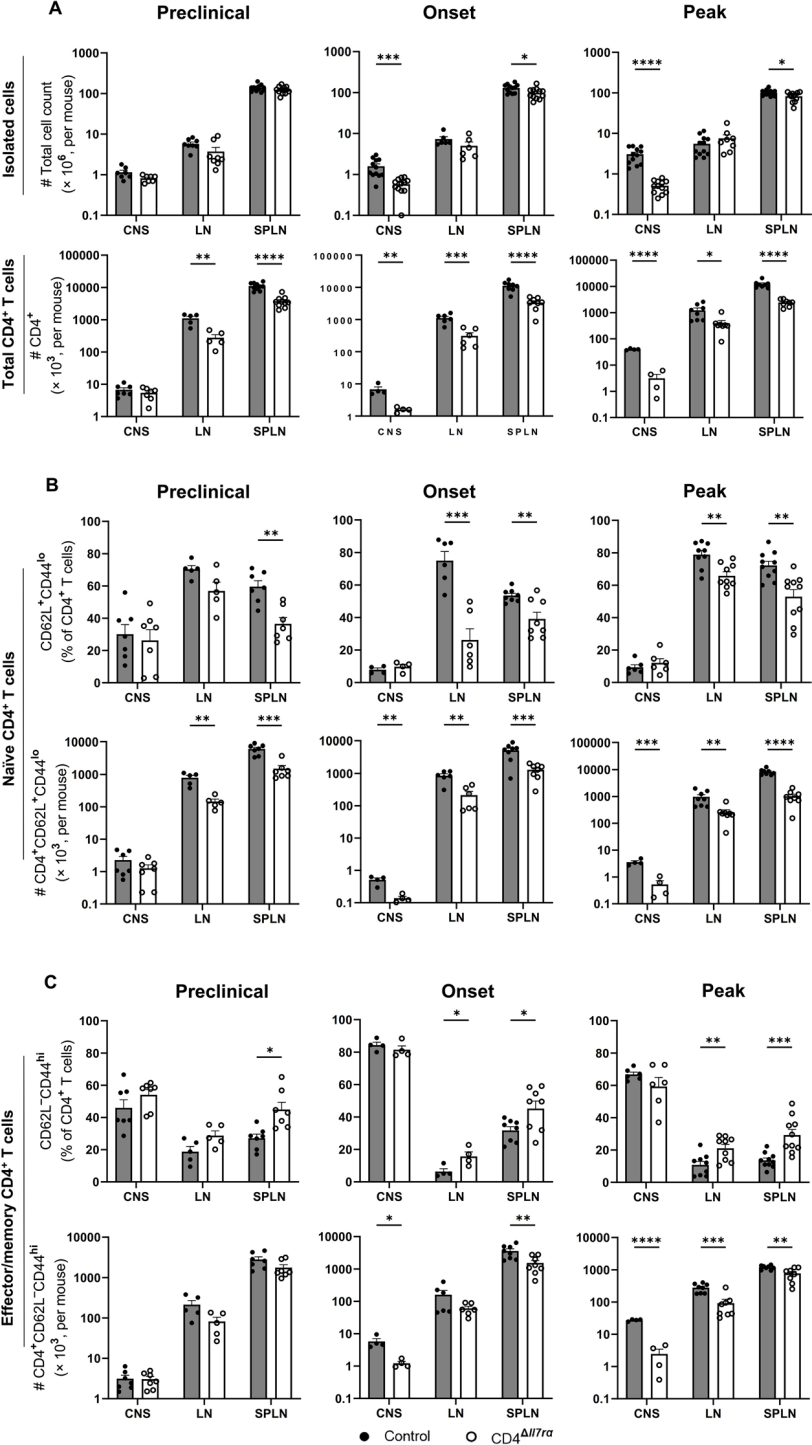
IL-7Rα gene knockout in CD4^+^ T cells reduces their numbers in immunized CD4^Δ*Il7ra*^ mice. Numbers of mononuclear cells (**A**) isolated from the CNS, LNs, and spleen of CD4^Δ*Il7ra*^ and CD4CreER^T2^ control mice at preclinical (8 d.p.i.), onset (12 d.p.i.), and peak (18 d.p.i.) of EAE in control mice. The frequencies and absolute numbers of total (**A**), naïve (**B**), and effector/memory (**C**) CD4^+^ T cells are also shown. Results are expressed as the mean ± SEM with *n* ≥ 5 per group from 2 independent experiments. Data were analyzed by Student’s t-test; *p < 0.05, **p < 0.01, ***p < 0.001

IL-7/IL-7R signaling plays a nonredundant role in the survival of naive CD4^+^ T cells and contributes to the homeostatic cycling of naive and memory CD4^+^ T cells [27]. Hence, it would be expected that IL-7Rα gene knockout in CD4^+^ T cells affects their numbers and characteristics. We examined these possibilities in mice immunized for EAE induction. We analyzed the spleen, LNs, and CNS of CD4^Δ*Il7ra*^ and control mice and found no difference in the numbers of isolated mononuclear cells between the two groups in the preclinical phase of EAE (8 d.p.i.), but the cell number obtained from the CNS of CD4^Δ*Il7ra*^ mice was lower at the time of onset and peak of the disease in control mice (Fig. 3A). The IL-7Rα gene knockout in CD4^+^ T cells decreased their numbers at preclinical, onset, and peak phase of EAE (in control mice) in the spleen and LNs, as well as at EAE onset and peak in the CNS (Fig. 3A). We found a similar decrease in absolute numbers of naïve and effector/memory CD4^+^ T cells (Fig. 3B and C). However, CD4^+^ T cells from CD4^Δ*Il7ra*^ mice showed an increase in the frequencies of effector/memory phenotype and a decrease in naïve CD4^+^ T cells compared to the control group (Fig. 3B and C). Overall, IL-7Rα gene knockout resulted in a progressive decline of CD4^+^ T cell numbers; at the time of immunization for EAE induction (5 d after starting tamoxifen treatment), numbers of CD4^+^ T cells in the spleen and LN of CD4^Δ*Il7ra*^ mice were reduced by 20%, and at EAE peak (23 d after starting tamoxifen treatment) by 80%. Total numbers and proportions of CD8^+^ T cells and CD19^+^ B cells were not affected in CD4^Δ*Il7ra*^ mice (data not shown).

Our data show that the IL-7Rα gene knockout in CD4^+^ T cells causes their depletion, starting soon after the knockout and progressing over the weeks to the extent that most of these cells were depleted. This phenomenon is not specific to immunized mice, as non-immunized mice also had reduced CD4^+^ T cell numbers (Fig. S2B). Our data indicate that the depleted state persists over 2–3 months due to slow repopulation. We tested to determine if CD4^Δ*Il7ra*^ mice that were depleted of CD4^+^ T cells by tamoxifen treatment and then allowed 3 months to fully repopulate would regain susceptibility to EAE. Indeed, these mice developed typical EAE (Fig. S5), demonstrating that the CD4^+^ T cell depletion did not have unexpected lasting consequences.

**Fig. 4:**
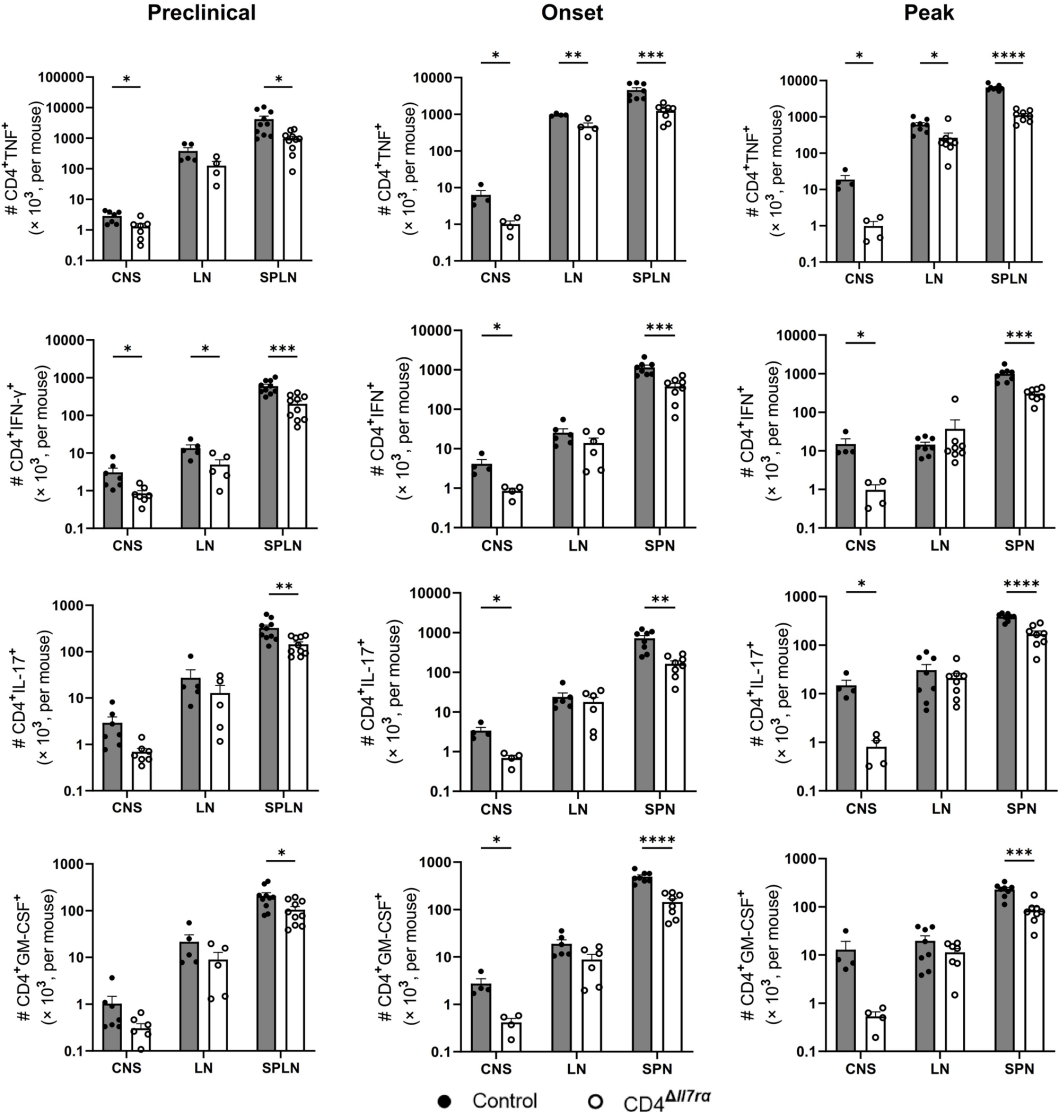
Fewer cytokine-producing Th cells are present in immunized CD4^Δ*Il7ra*^ mice. Numbers of cytokine-producing CD4^+^ T cells from the spleen, LNs, and CNS of CD4^Δ*Il7ra*^ and the control mice during EAE. Results are expressed as the mean ± SEM with *n* ≥ 4 per group from 2 independent experiments. Data were analyzed by Student; s t-test; *P < 0.05; **P < 0.01; ***P < 0.001; ****P < 0.0001

To examine whether the IL-7Rα gene knockout in CD4^+^ T cells alters the composition of Th lineages among remaining cells, we evaluated the proportion and absolute number of TNF-, IFN-γ-, IL-17 A-, and GM-CSF-expressing CD4^+^ T cells in the spleens, LNs, and CNS of immunized mice. Compared to the control group, there was an increase in the percentage of CD4^+^ T cells expressing IFN-γ, IL-17, and GM-CSF in the spleens and LNs of CD4^Δ*Il7ra*^ mice at the time of EAE onset and peak in control mice (Fig. S6, Fig. S7A and B). However, the total numbers of TNF-, IFN-γ-, IL-17 A-, and GM-CSF-producing CD4^+^ T cells decreased in the spleens and CNS of CD4^Δ*Il7ra*^ mice compared to control mice at the onset and peak of disease (Fig. 4). We found no significant difference in IL-10-producing cells between the two groups (Fig. S8A). Mean fluorescence intensity (MFI) for TNF, IL-17, GM-CSF, and IL-10 in CD4^+^ T cells from the spleens remained the same among the cells that produced these cytokines, except for IFN-γ (Fig. S8B), indicating that fewer cytokine-producing cells are available in the CD4^+^ T cell pool of CD4^Δ*Il7ra*^ mice, and this is not directly related to lack of IL-7Rα signaling in this effector cells.

To test if impaired migratory capacity of remaining CD4^+^ T cells in CD4^Δ*Il7ra*^ mice could be contributing to their EAE resistance, we assessed the expression of chemokine receptors CCR6, CXCR5, and CXCR6, which play key roles in CD4^+^ T cell migration and EAE pathogenesis [28, 29]. We found an increased percentage of CD4^+^Foxp3^−^ T cells expressing CCR6 and CXCR6, but not CXCR5, in the spleens and lymph nodes of CD4^Δ*Il7ra*^ mice compared to control mice in the preclinical phase of EAE (Fig. S9). The greater percentages of CCR6^+^ and CXCR6^+^ CD4^+^ T cells could be attributed to the increased proportion of effector/memory CD4^+^ T cells [28] that remain in CD4^Δ*Il7ra*^ mice after tamoxifen treatment (Fig. 3C). However, because of the overall loss of CD4^+^ T cells in CD4^Δ*Il7ra*^ mice, the total numbers of CD4^+^ T cells expressing the chemokine receptors were reduced despite their increased proportions. These data suggest that the remaining Th cells in CD4^Δ*Il7ra*^ mice do not have a defect in migratory capacity and that such a defect is not a contributing factor to the EAE resistance of these mice.

**Fig. 5:**
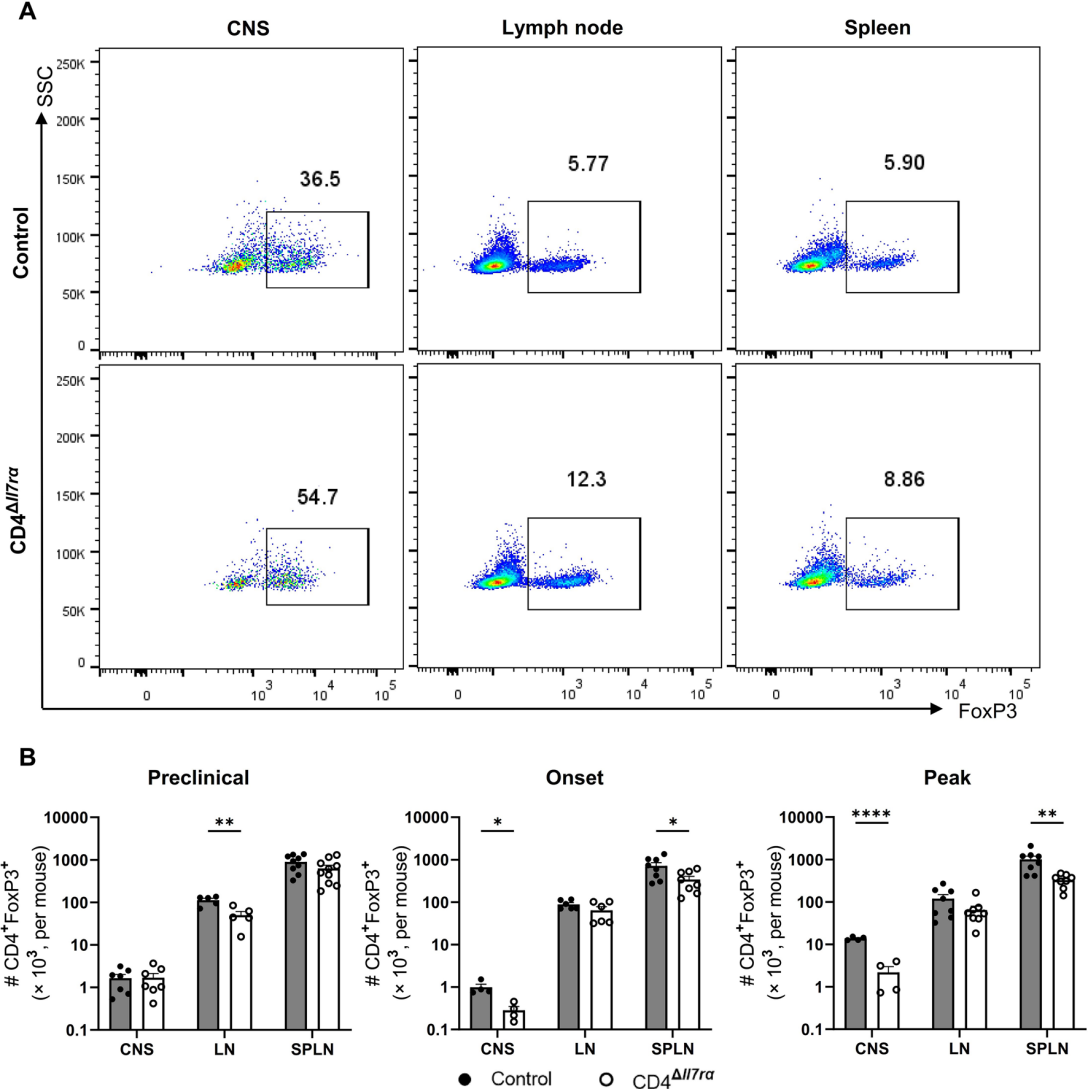
Increased proportions of Treg cells among CD4^+^ T cells of CD4^Δ*Il7ra*^ mice. (**A**) Representative flow cytometry plots of CD4^+^Foxp3^+^ cells in gated CD4^+^ T cells at EAE peak in CD4^Δ*Il7ra*^ and control mice. (**B**) A total number of CD4^+^Foxp3^+^ T cells in the CNS, LNs, and spleen of CD4^Δ*Il7ra*^ mice compared with control mice at different time points during EAE. Results are expressed as the mean ± SEM with *n* ≥ 4 per group from 3 independent experiments and were analyzed by Student’s t-test; *P < 0.05; **P < 0.01; ****P < 0.0001

Given that Treg cells express low levels of IL-7Rα and are frequently characterized as IL-7Rα^low^^/^^−^, we examined how IL-7Rα gene knockout in CD4^+^ T cells impacts Tregs. The frequency of CD4^+^Foxp3^+^ T cells among CD4^+^ T cells in the CNS and peripheral lymphoid tissue was higher in CD4^Δ*Il7ra*^ mice than in control mice (Fig. S10A and Fig. 5A). However, total numbers of CD4^+^Foxp3^+^ cells in immunized CD4^Δ*Il7ra*^ mice either decreased or remained similar compared to control mice (Fig. 5B). A similar pattern was observed when we evaluated CD4^+^Foxp3^+^RORγt^+^ Treg cells during the preclinical phase of EAE (Fig. S10B and C) and when Treg cells were defined as CD4^+^CD25^+^Foxp3^+^(Fig. S10D). These data show that, upon IL-7Rα gene knockout, the numbers of Treg cells decline less compared to those of other CD4^+^ T cells, but their total numbers also tend to be reduced.

### CD4^Δ*Il7ra*^ mice develop diminished MOG-specific CD4^+^ T cell response

**Fig. 6:**
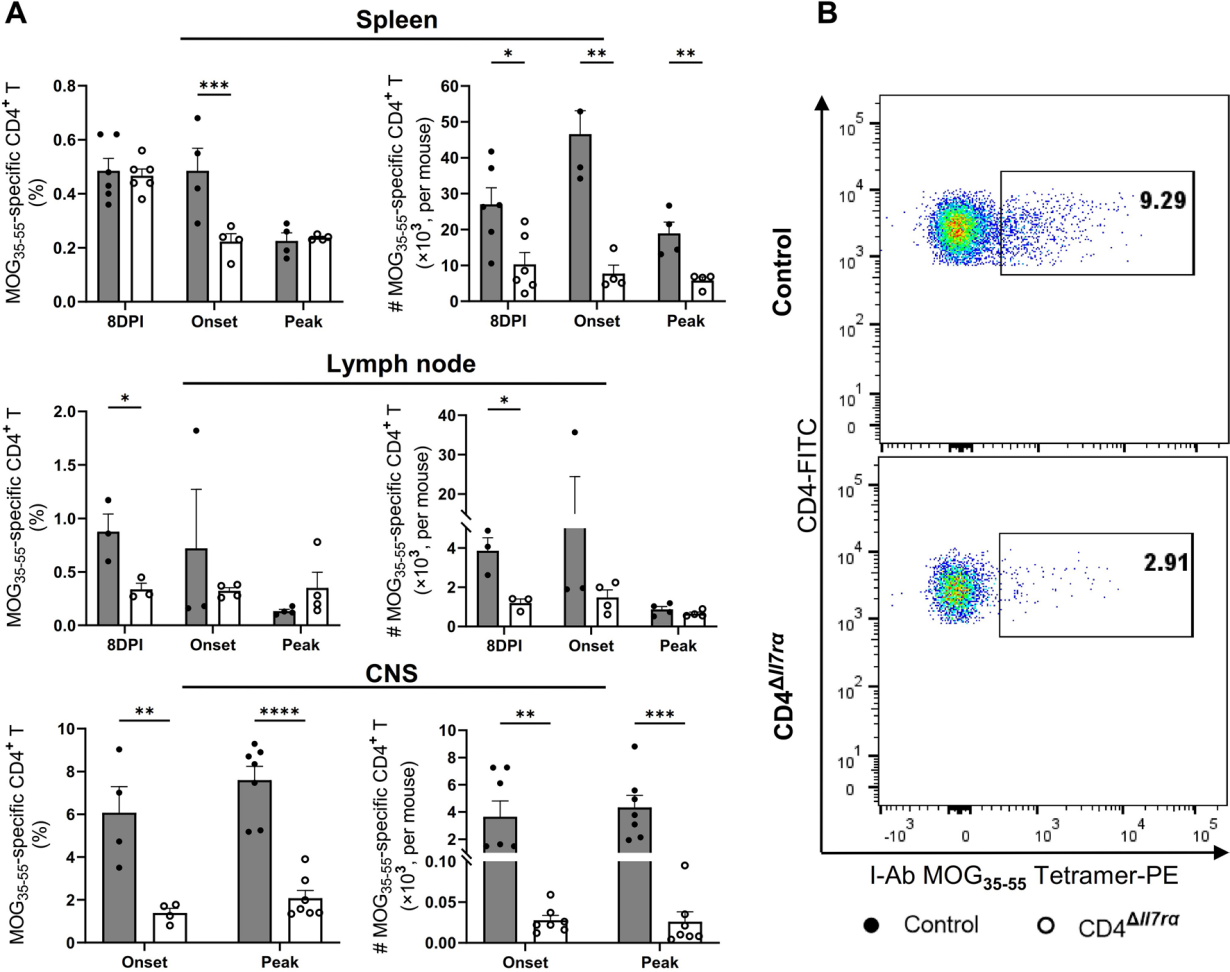
IL-7Rα gene knockout in CD4^+^ T cells abrogates MOG-specific response. Spleen, LNs, and CNS cells from MOG_35 − 55_-immunized CD4^Δ*Il7ra*^ and control mice were harvested and stained with I-Ab MOG_35 − 55_ MHC tetramer-PE and stained for CD4 and CD45 and analyzed by flow cytometry. (**A**) The frequencies of MOG_35 − 55_-specific CD4^+^ T cells in total CD4^+^ T cells and absolute numbers of MOG_35 − 55_-specific CD4^+^ T cells are shown. Results are expressed as the mean ± SEM with *n* ≥ 3 per group from 2 independent experiments. (**B**) Dot plots from the CNS of one representative experiment are shown (percentage of tetramer^+^ cells within the CD45^+^CD4^+^ T cells)

IL-7/IL-7R signaling plays an important role in the development of myelin-specific CD4^+^ T cell response during EAE [16, 30]. To characterize the effect of IL-7Rα gene knockout in CD4^+^ T cells on the development of MOG-specific response in our model, we examined the frequency and function of MOG_35 − 55_-specific CD4^+^ T cells in immunized CD4^Δ*Il7ra*^ mice. Spleen, LN, and CNS cells were stained with MOG_35 − 55_/IAb tetramers and restimulated with MOG_35 − 55_ peptide to measure proliferation and cytokine production. Tracking MOG_35 − 55_-specific CD4^+^ T cells with the tetramer showed a large reduction in the numbers of tertramer^+^ CD4^+^ T cells from CD4^Δ*Il7ra*^ mice, especially in the spleen and CNS at the onset and peak of EAE in control mice (Fig. 6A and B).

**Fig. 7:**
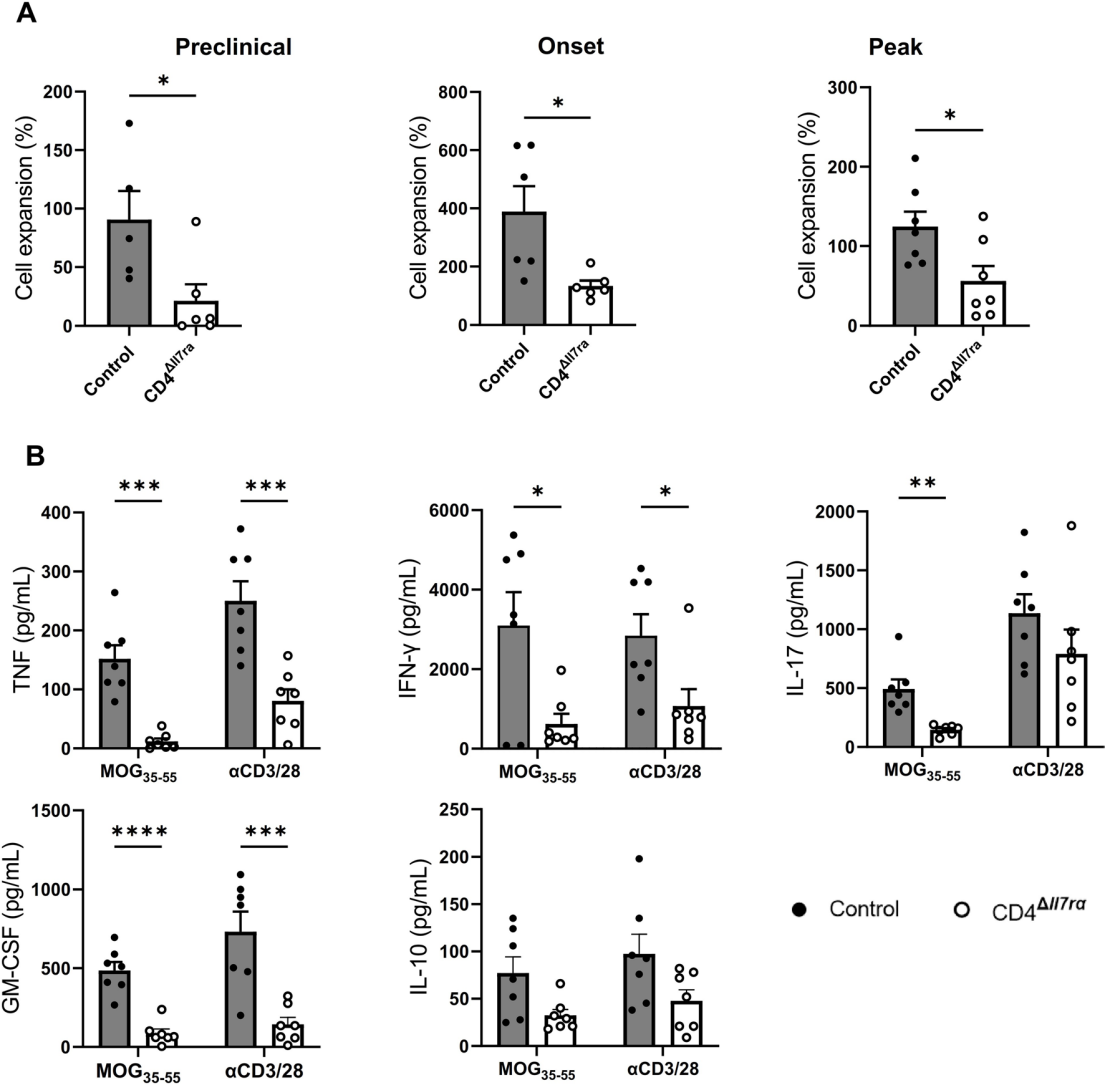
Diminished MOG_35 − 55_-specific CD4^+^ T cell response in CD4^Δ*Il7ra*^ mice. For cytokine measurement and proliferation assay, splenocytes were seeded (2.5 × 10^5^ cells per well) in a 96-well plate and activated with MOG_35 − 55_. Parallel samples were activated with anti-CD3 and anti-CD28 mAbs. (**A**) After four days, cell numbers were elucidated by XTT assay. The proliferation of MOG_35 − 55_-stimulated cells is represented as a percentage of the proliferation of a corresponding sample activated with anti-CD3 and anti-CD28 mAbs. (**B**) Cytokine concentrations were measured by ELISA in the culture supernatants at the onset of EAE in control mice. Results are expressed as the mean ± SEM for triplicate cultures of splenocytes from 2 independent experiments (*n* = 7) and were analyzed by Student; s t-test; *P < 0.05; **P < 0.01; ***P < 0.001; ****P < 0.0001

To further characterize MOG_35 − 55−_specific CD4^+^ T cell response in CD4^Δ*Il7ra*^ mice, we tested proliferative and cytokine recall responses to MOG_35 − 55_. The proliferative responses of CD4^Δ*Il7ra*^ splenocytes were markedly decreased compared with those of control mice (Fig. 7A). Similarly, splenocytes of CD4^Δ*Il7ra*^ mice secreted less TNF, GM-CSF, IFN-γ, and IL-17 compared to the control, but we found no differences in the levels of IL-10 (Fig. 7B). Reduced cytokine production by the cells from CD4^Δ*Il7ra*^ mice could be explained by diminished MOG_35 − 55_-specific CD4^+^ T cell response. However, it is possible that the lack of IL-7Rα signaling in existing MOG_35 − 55_-specific CD4^+^ T cells from CD4^Δ*Il7ra*^ mice contributed to their reduced cytokine production. These results support the possibility that CD4^Δ*Il7ra*^ mice do not develop EAE because of the markedly diminished magnitude of their MOG-specific CD4^+^ T cell response.

Next, we assessed proliferation of CD4^+^ T cells in the peripheral lymphoid tissue of CD4^Δ*Il7ra*^ mice during the preclinical phase of EAE by staining for Ki-67. We observed a higher frequency of Ki-67^+^CD4^+^Foxp3^−^ T cells in the spleen and LNs of CD4^Δ*Il7ra*^ mice compared to control mice; however, their total numbers were reduced. In contrast, there was no difference in the frequency of Ki-67^+^CD4^+^Foxp3^+^ Treg cells, but their total number was also reduced in CD4^Δ*Il7ra*^ mice compared to control mice (Fig. S11A and B). Effector/memory CD4^+^ T cells typically express higher levels of Ki-67 compared to naive CD4^+^ T cells, as they are more likely to be in an active or recently proliferated state. In contrast, naive CD4^+^ T cells are usually in a resting state and, therefore, express lower levels of Ki-67 [31, 32].

## Discussion

Certain polymorphisms in the IL-7Rα gene have been reported as a risk factor for MS [18, 20, 21]. Given that IL-7Rα plays a major role in the biology of CD4^+^ T cells, which are regarded as primary drivers of MS immunopathology, understanding how IL-7Rα impacts CD4^+^ T cells in the context of CNS autoimmunity is important. To this end, we used conditional knockout of the IL-7Rα gene in CD4^+^ T cells in the mouse EAE model. We knocked out the IL-7Rα gene in adult mice, circumventing gross developmental defects of the immune system present in mice with constitutive knockout of the IL-7Rα gene in CD4^+^ T cells. Hence, using this model, we examined the role of IL-7Rα in CD4^+^ T cells within the normal immune system of adult mice in steady state and EAE. After the tamoxifen levels in treated mice tapered off (within several days), the normal thymopoiesis and seeding of the periphery with new IL-7Rα^+^ T cells could resume. Our main findings from this model are: (a) IL-7Rα^+^ on peripheral CD4^+^ T cells was necessary for EAE development; (b) the loss of the IL-7Rα gene in CD4^+^ T cells led to a gradual and substantial decline in their numbers; (c) Treg cell number declined as well, but less than other CD4^+^ T cells; (d) repopulation of CD4^+^ T cell pool at the periphery was slow; and (e) all naive CD4^+^ T cells became IL-7Rα^−^ after tamoxifen treatment, but a portion of effector/memory CD4^+^ T cells remained IL-7Rα^+^.

CD4^Δ*Il7ra*^ mice did not develop CNS inflammation or clinical signs of EAE, even though, at the time of immunization, these mice had numbers of CD4^+^ T cells similar to control mice. Their numbers in CD4^Δ*Il7ra*^ mice declined up to 75%, 8 days after immunization compared to control mice. MOG-specific CD4^+^ T cell response in CD4^Δ*Il7ra*^ mice was also diminished, as determined by staining with tetramer, MOG-induced cytokine production, and proliferation in vitro. It is clear that CD4^+^ T cells without IL-7Rα cannot mount an effective response and that repopulation with new IL-7Rα^+^ cells is too slow to compensate for the disappearance of IL-7Rα^−^ cells. This agrees with previous reports that IL-7Rα^−/−^ mice are resistant to EAE [26]. Accordingly, short-term IL-7Rα blockade with mAb inhibited autoantigen-specific CD4^+^ T cell response and prevented and ameliorated disease [19, 21, 30]. Interestingly, blockade of IL-7Rα after EAE onset also suppressed disease, demonstrating that IL-7Rα is required not only for developing CD4^+^ T cell response but also for its maintenance [19, 21, 30]. However, in these studies, IL-7Rα was either knocked out or blocked systemically, and observed effects cannot be attributed solely to the role of IL-7Rα in CD4^+^ T cells.

Ashbaugh et al. addressed the role of IL-7Rα in CD4^+^ T cells in EAE more specifically; in their studies, mice lacking IL-7Rα in hematopoietic cells developed typical EAE, suggesting that IL-7Rα expressed by non-hematopoietic cells was sufficient for severe disease to develop [19]. This starkly contrasts with our result that IL-7Rα gene knockout in CD4^+^ T cells abrogates EAE development. In our model, IL-7Rα sufficiency in nonhematopoietic and most hematopoietic cells did not compensate for IL-7Rα deficiency in CD4^+^ T cells. Our results are consistent with numerous reports that IL-7Rα deficiency in CD4^+^ T cells results in notably diminished immune responses [13, 26, 30], which would, therefore, be expected to attenuate EAE, as happened in our model. Our data clearly show that IL-7Rα expression in any cell type other than CD4^+^ T cells is irrelevant in developing B cell-independent CD4^+^ T cell-driven EAE. Further, Ashbaugh et al. used IL7RTg^IL7R−/−^ mice, which were engineered to express IL-7Rα in the thymus of IL-7Rα^−/−^ mice [33], enabling T cell development in the thymus and seeding the periphery; however, these T cells ceased to express IL-7Rα in the periphery. Naïve IL7RTg^IL7R−/−^ mice had normal numbers of (IL-7Rα^−^) CD4^+^ T cells in the spleen, while numbers of (IL-7Rα^−^) CD8^+^ T cells were reduced by ~ 50%, and B cells by ~ 85%. Once again, this contrasts with our results that IL-7Rα deficiency in peripheral CD4^+^ T cells causes a profound loss of these cells from the periphery. We have seen up to an 80% reduction in splenic CD4^+^ T cells one month after tamoxifen treatment. Our results are consistent with abundant literature that maintaining the CD4^+^ T cell pool in the periphery requires IL-7Rα [34–36]. Thus, we do not have an explanation for the discrepant findings between Ashbaugh et al. and our group.

It has been well documented that IL-7Rα has a pivotal role in developing, surviving, and maintaining naive and effector/memory CD4^+^ T cells [34, 35], and our results are consistent with these reports. In our model, effector/memory CD4^+^ T cells were relatively resistant to downregulation of IL-7Rα compared to naïve cells. We did not determine the efficacy of knocking out the IL-7Rα gene, so we do not know if effector/memory and naïve CD4^+^ T cells differ in the extent of the gene knockout, which could explain the difference in the extent of downregulation at the cell surface. Possible reasons for reduced knockout of the IL-7Rα gene in the effector/memory subpopulation could be lower Cre levels if there is less CD4 promoter-driven expression of Cre in this cell than in naïve cells. Alternatively, both naïve and effector memory cells had similar levels of IL-7Rα gene knockout, but the latter maintained IL-7Rα protein presence on the cell surface for a longer time than naïve cells. Consistent with the phenomenon that a portion of effector/memory cells remained IL-7Rα^+^, unlike naïve cells, we found an increase over time in the proportion of effector/memory CD4^+^ T cells among CD4^+^ T cells of CD4^Δ*Il7ra*^ mice relative to control mice. This increase in the proportion of effector/memory cells could be driven by enhanced proliferation and survival of remaining IL-7Rα^+^ effector/memory CD4^+^ T cells compared to naïve cells, which were virtually all IL-7Rα^−^. Alternatively, survival/proliferation of even IL-7Rα^−^ effector/memory cells could be independent of IL-7Rα, as described [37]. Effector/memory CD4^+^ T cells have likely adapted to rely on multiple survival signals beyond IL-7Rα [38]. This redundancy ensures their persistence even if one signaling pathway is disrupted. They might have alternative mechanisms that compensate for the loss of IL-7Rα.

IL-7Rα signaling plays a crucial role in the survival of naïve CD4^+^ T cells and the homeostatic proliferation of memory CD4^+^ T cells. Tan et al. reported that naive T cells proliferated minimally in IL-7^−/−^ mice. Apart from homeostatic proliferation, the extended survival of naive T cells relies on IL-7/IL-7R signaling. As a result, naive T cells gradually diminished over one month following their transfer into IL-7^−/−^ mice. These findings suggest that naive CD4^+^ T cells depend on IL-7/IL-7R for survival and homeostatic proliferation [36], but the homeostatic proliferation of effector/memory CD4^+^ T cells is independent of IL-7/IL-7R signaling [37].

In the current study, attenuation of EAE in the CD4^Δ*Il7ra*^ mice was associated with a decrease in CD4^+^ T cell responses in the CNS and lymphoid organs. Although there was no significant reduction in the number of CD4^+^ T cells in lymphoid organs of CD4^Δ*Il7ra*^ mice at the time of immunization, IL-7Rα deletion in CD4^+^ T cells resulted in a gradual decrease in their numbers during the course of EAE. Our results revealed that an absence of IL-7Rα signaling diminished the frequency and function of MOG_35 − 55_-specific CD4^+^ T cells. We showed that CD4^Δ*Il7ra*^ mice had fewer MOG_35 − 55_-specific CD4^+^ T cells at the onset and peak of EAE. In addition, the proliferative responses of MOG_35 − 55_ stimulated CD4^+^ T cells derived from CD4^Δ*Il7ra*^ mice were markedly decreased. The number of cytokine-producing CD4^+^ T cells was also decreased in CD4^Δ*Il7ra*^ EAE mice. Similarly, MOG_35–55_ re-stimulated splenocytes from CD4^Δ*Il7ra*^ mice produced less cytokines. This is consistent with other studies, as Walline et al. reported that the reduced secretion of inflammatory cytokines in IL-7Rα^−/−^ mice is attributable to a lower count of CD4^+^IL-17^+^ and CD4^+^IFN-γ^+^ splenocytes, as well as impaired Th1 cell differentiation [26]. Ashbaugh et al. reported that spinal cord infiltrating cells from IL-7Rα deficient EAE mice had fewer TNF- and IFN-γ-producing CD4^+^ T cells. During the acute phase of the disease, Th17 cells were either minimal or absent in the spleen and spinal cord [19]. Accordingly, when used to treat EAE, short-term in vivo IL-7Rα blockade hindered the activation and expansion of CD4^+^ T cells specific to autoantigen and prevented and ameliorated disease [30]. Our results confirmed that the EAE resistance of CD4^Δ*Il7ra*^ mice is caused by insufficient CD4^+^ T cell sensitization against the MOG_35 − 55_ peptide due to intrinsic deficiency of IL-7R^−/−^ CD4^+^ T cells.

In our model, the loss of IL-7Rα in CD4^+^ T cells resulted in greater frequency (~ 2x) of Treg cells among CD4^+^ T cells, but total numbers of Treg cells were notably reduced (by up to 66%) compared to control mice. The greater frequency of Tregs could be explained by the lower rate of their decline compared to other CD4^+^ T cells that express markedly more IL-7Rα and are more acutely dependent on it for their survival and proliferation. Treg cells in the periphery express very little IL-7Rα [39], which could be considered functionally inconsequential [40]. However, studies have reported that activated Treg cells upregulate IL-7Rα expression [41], which contrasts with other CD4^+^ T cells that temporarily downregulate IL-7Rα upon activation. Further, it has been shown that IL-7Rα in peripheral Tregs is required for their proliferation/survival and optimal suppressive function [42]. These effects are mediated *via* increased CD25 expression and enhanced IL-2 signaling in the presence of IL-7Rα on Treg cells [42, 43]. Hence, one reason for the decline of Treg cell numbers in CD4^Δ*Il7ra*^ mice could be reduced survival and proliferation of Treg cells due to their intrinsically reduced responsiveness to IL-2 in the absence of IL-7Rα. This could be compounded by reduced availability of IL-2 in their milieu because of decreased numbers of CD4^+^ T cells and diminished immune responses in CD4^Δ*Il7ra*^ mice. Another reason for the decline of Treg cell numbers in CD4^Δ*Il7ra*^ mice could be the reduced output of *de novo* developed Treg cells from the thymus [10]. Although we treated CD4^Δ*Il7ra*^ mice with tamoxifen only once or twice, we cannot exclude the possibility that its negative effect on IL-7Rα expression by thymocytes and the development of naïve CD4^+^ T cells, including Treg cells, could be relatively long-lasting, which contributes to the decline of CD4^+^ T cell numbers and of Treg cells among them. This possibility of tamoxifen’s long-lasting direct suppressive effect is consistent with a prolonged period (~ 3 months) needed for CD4^+^ T cells to repopulate the periphery fully. However, a long-lasting effect of tamoxifen would also decrease the thymic output of CD8^+^ T cells [10], eventually manifesting as a diminished CD8^+^ T cell population in the periphery. We did not find a decline in the CD8^+^ T cell population, which contradicts the possibility of prolonged suppression of the thymic output of T cells, including Treg cells. Hence, if the thymic output is not suppressed for a prolonged time, then the decline in Treg cell numbers is likely due to the loss of IL-7Rα on peripheral Treg cells and, possibly, because of the depletion of CD4^+^ T cells as a source of IL-2. However, we did not determine if IL-2 levels in tamoxifen-treated CD4^Δ*Il7ra*^ mice are lower than in control mice. To answer this question, the thymic output of T cells post-tamoxifen treatment would need to be characterized. This would also begin to answer whether the slow recovery of CD4^+^ T cell niche is caused by retarded thymic output, possibly due to long-lasting tamoxifen effects, or because thymopoiesis is an intrinsically slow process, at least in adult mice. It has been reported that the depletion of CD4^+^ T cells with mAb resulted in the complete absence of CD4^+^ T cells for at least a month and that their numbers recovered to ~ 80% of normal numbers 2 months after the depletion [44]. In two non-human primates, recovery of CD4^+^ T cells after depletion with mAb was slow, reaching only 50% at 6 months after the depletion [45]. Of note, in the same study, the repopulation of CD8^+^ T cells was markedly faster and more complete than that of CD4^+^ T cells [45]. Similarly, in humans who received hematopoietic stem cell transplants, the reconstitution of CD4^+^ T cells was much slower than that of CD8^+^ T cells [46, 47]. In the studies wherein alemtuzumab treatment depleted CD4^+^ and CD8^+^ T cells, patients had profound and long-lasting depletion of T cells, with reconstitution reaching only 50% of normal values 3 years post depletion. The recovery of CD4^+^ T cells was slower and not as thorough as that of CD8^+^ T cells [48, 49].

The response of CD4^+^ T cells to TSLP signaling is mediated by the TSLPR complex, which consists of IL-7Rα and TSLPR [1]. Due to the shared IL-7Rα subunit involved in both IL-7 and TSLP signaling pathways [2], the effects of IL-7Rα knockout in CD4^+^ T cells reflect the loss of signaling from both IL-7 and TSLP. However, the effects of IL-7 are notably more dramatic than those of TSLP [1, 50], indicating that the phenotype of CD4^Δ*Il7ra*^ mice is primarily caused by the lack of IL-7 signaling, although the lack of TSLP signaling may be a contributing factor. It has been shown that mice lacking TSLP or TSLP-specific receptor subunit (whole-body knockouts) develop mildly ameliorated EAE disease compared to control mice [28, 51]. This is consistent with, but notably less profound than, the complete resistance of CD4^Δ*Il7ra*^ mice to EAE that we have observed. This indicates that the phenotype of CD4^Δ*Il7ra*^ mice, including EAE resistance, is substantially attributable to the lack of IL-7 signaling.

In summary, our study demonstrates that IL-7Rα on peripheral CD4^+^ T cells is indispensable for their survival and function. Loss of IL-7Rα on peripheral CD4^+^ T cells results in their depletion and inability to mount an immune response upon challenge. In EAE, this manifests as a complete resistance to disease induction, which underscores that IL-7Rα on CD4^+^ T cells is essential for EAE to develop.

## Electronic supplementary material

Below is the link to the electronic supplementary material.

Supplementary Material 1

Supplementary Material 2

Supplementary Material 3

Supplementary Material 4

Supplementary Material 5

Supplementary Material 6

Supplementary Material 7

Supplementary Material 8

Supplementary Material 9

Supplementary Material 10

Supplementary Material 11

Supplementary Material 12

Supplementary Material 13

## Data Availability

No datasets were generated or analysed during the current study.
