# Supplementary figures and images for: IL-7Rα on CD4+ T cells is required for their survival and the pathogenesis of experimental autoimmune encephalomyelitis
Source: J Neuroinflammation. 2024 Oct 8;21:253. doi: 10.1186/s12974-024-03224-2 (PMC11460225; doi:10.1186/s12974-024-03224-2)

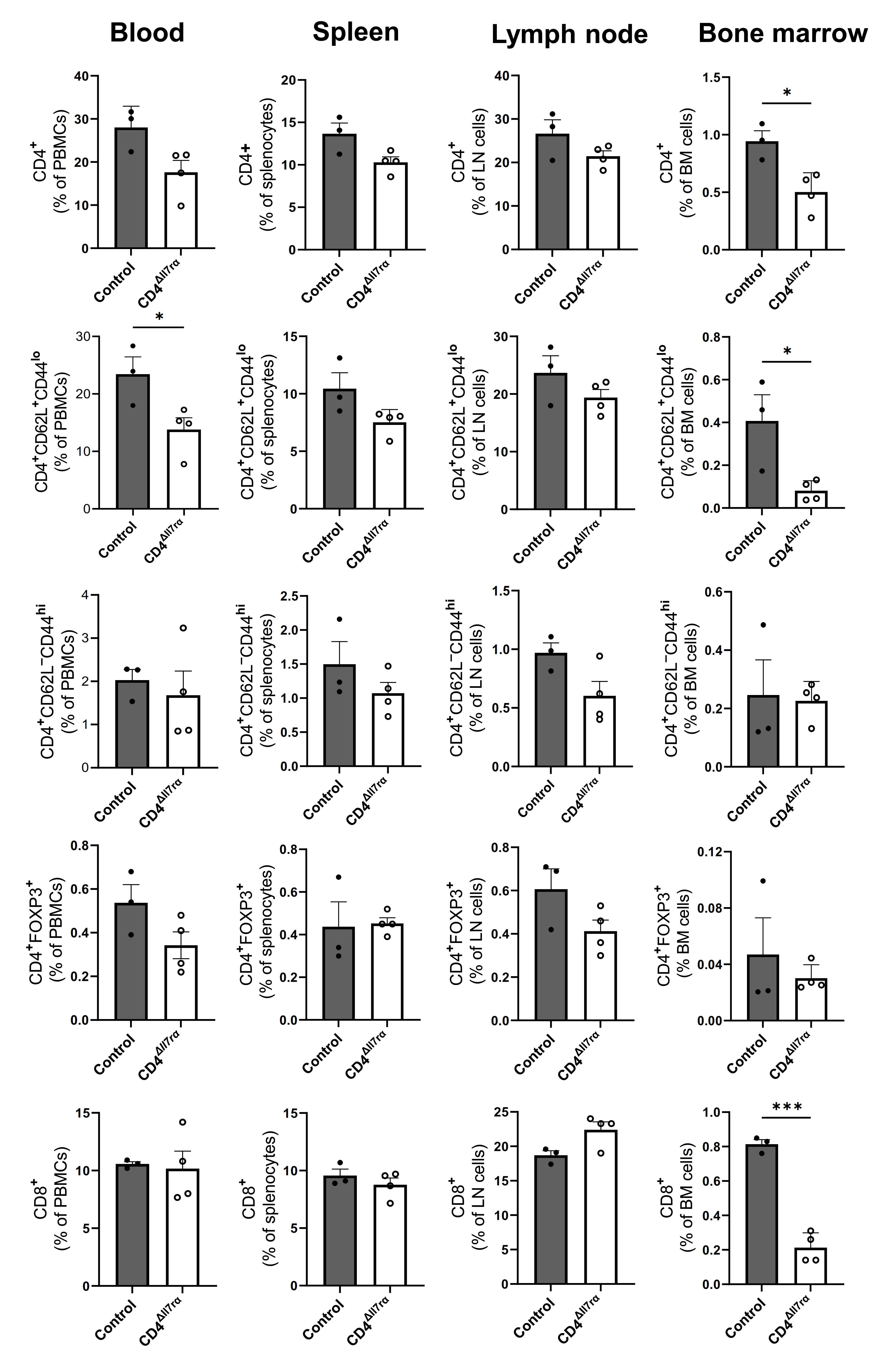

Supplement: Supplementary file 1 — Supplementary Material 1 [file 12974_2024_3224_MOESM1_ESM.jpg]

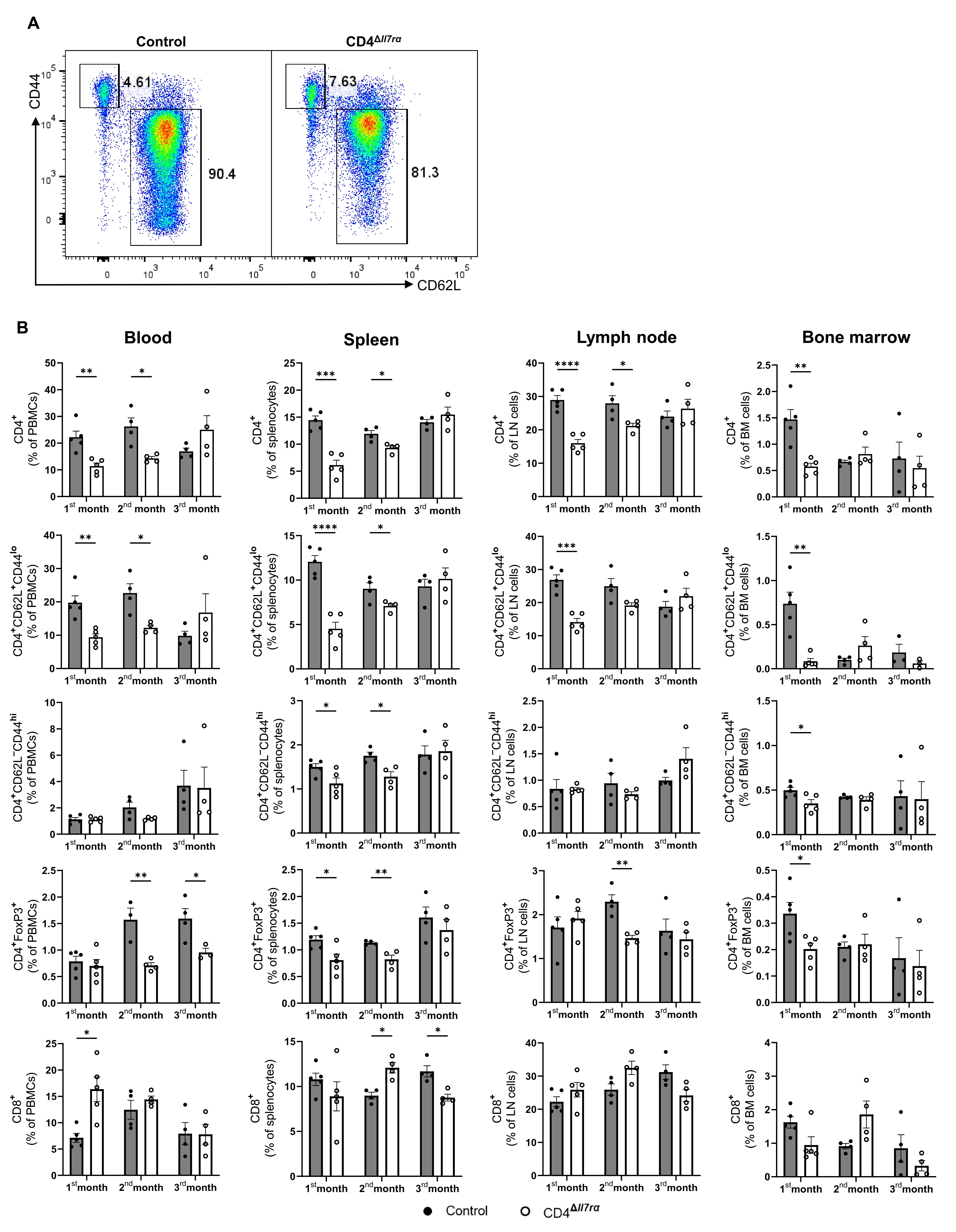

Supplement: Supplementary file 2 — Supplementary Material 2 [file 12974_2024_3224_MOESM2_ESM.jpg]

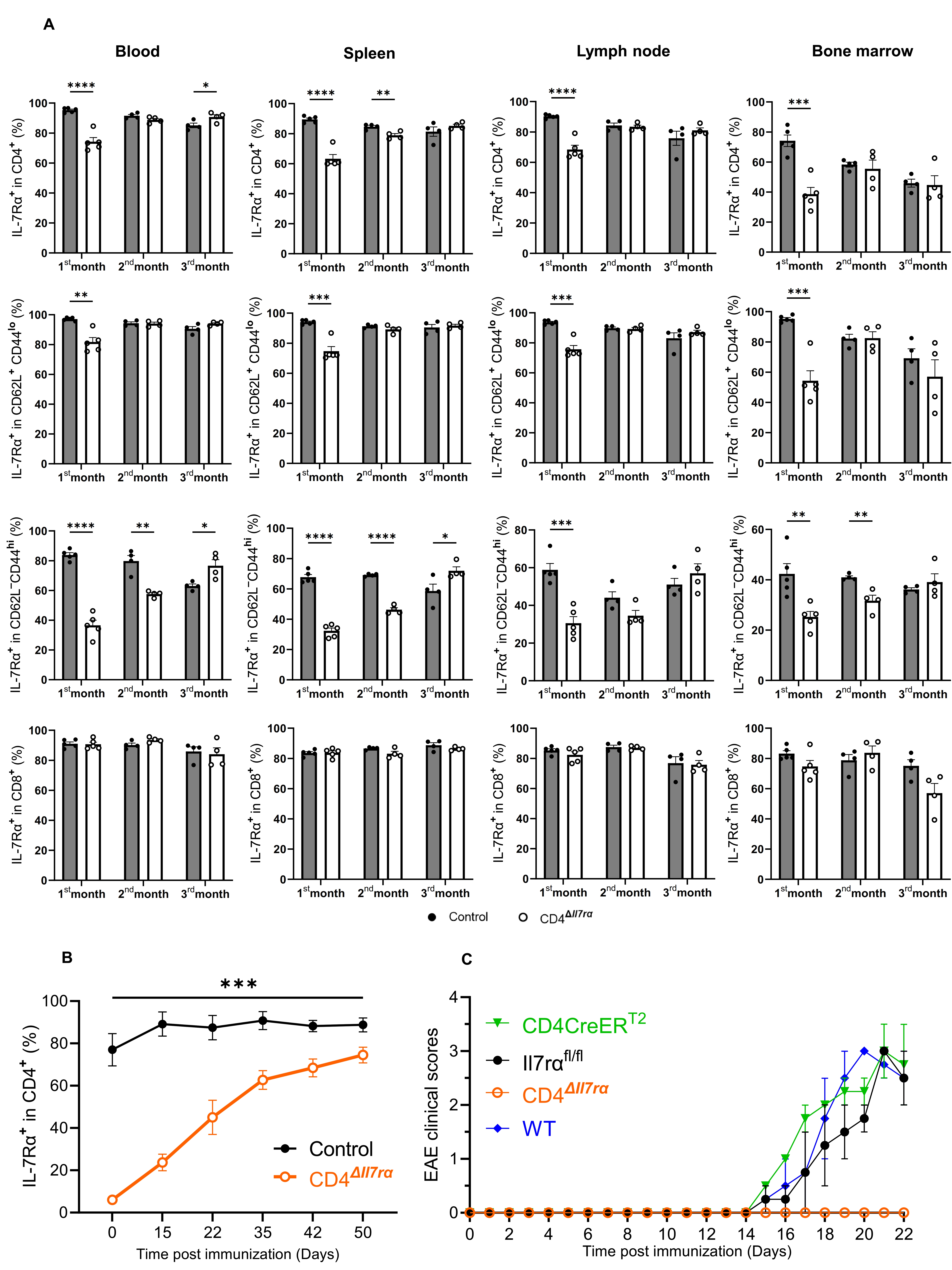

Supplement: Supplementary file 3 — Supplementary Material 3 [file 12974_2024_3224_MOESM3_ESM.jpg]

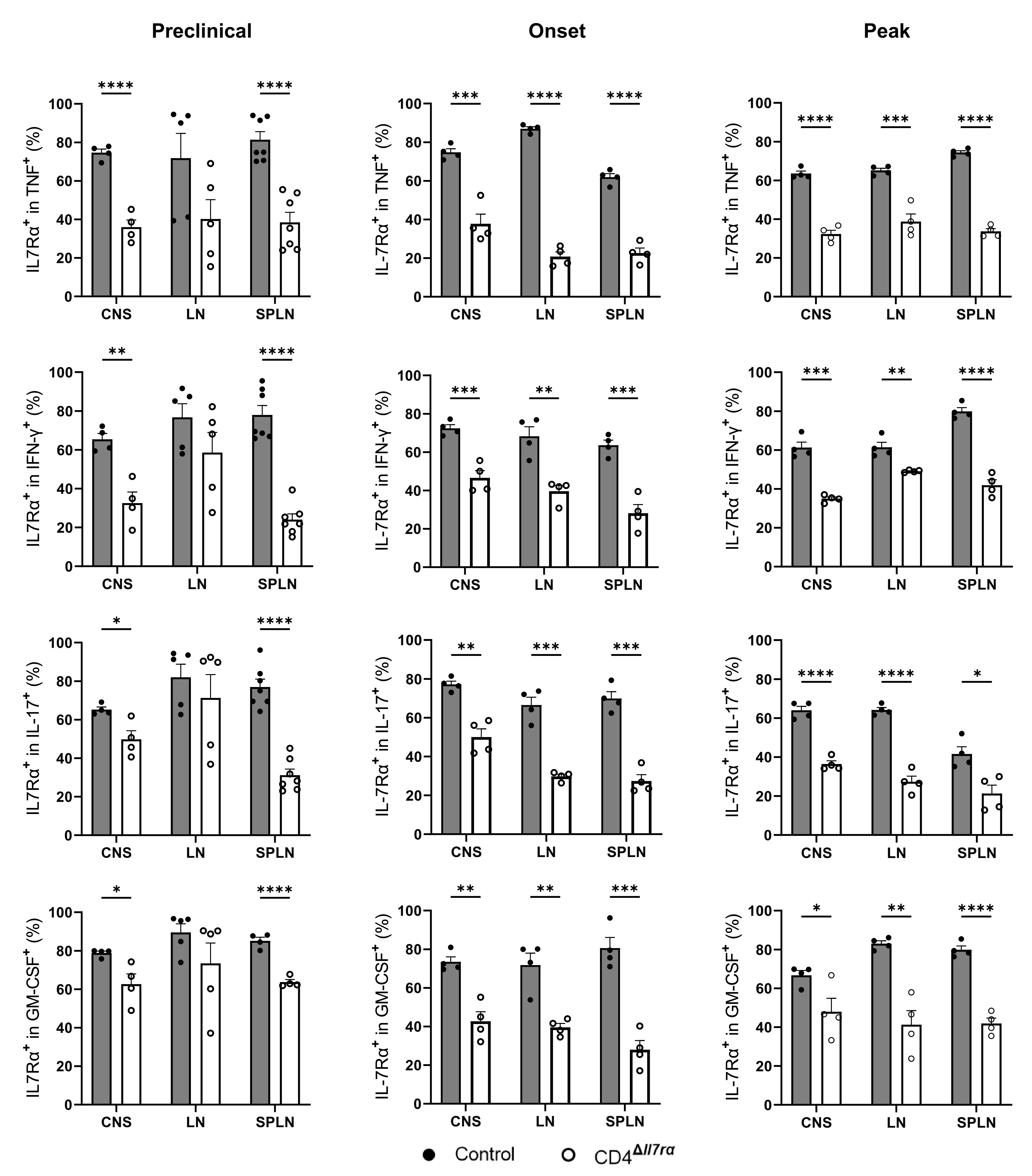

Supplement: Supplementary file 4 — Supplementary Material 4 [file 12974_2024_3224_MOESM4_ESM.jpg]

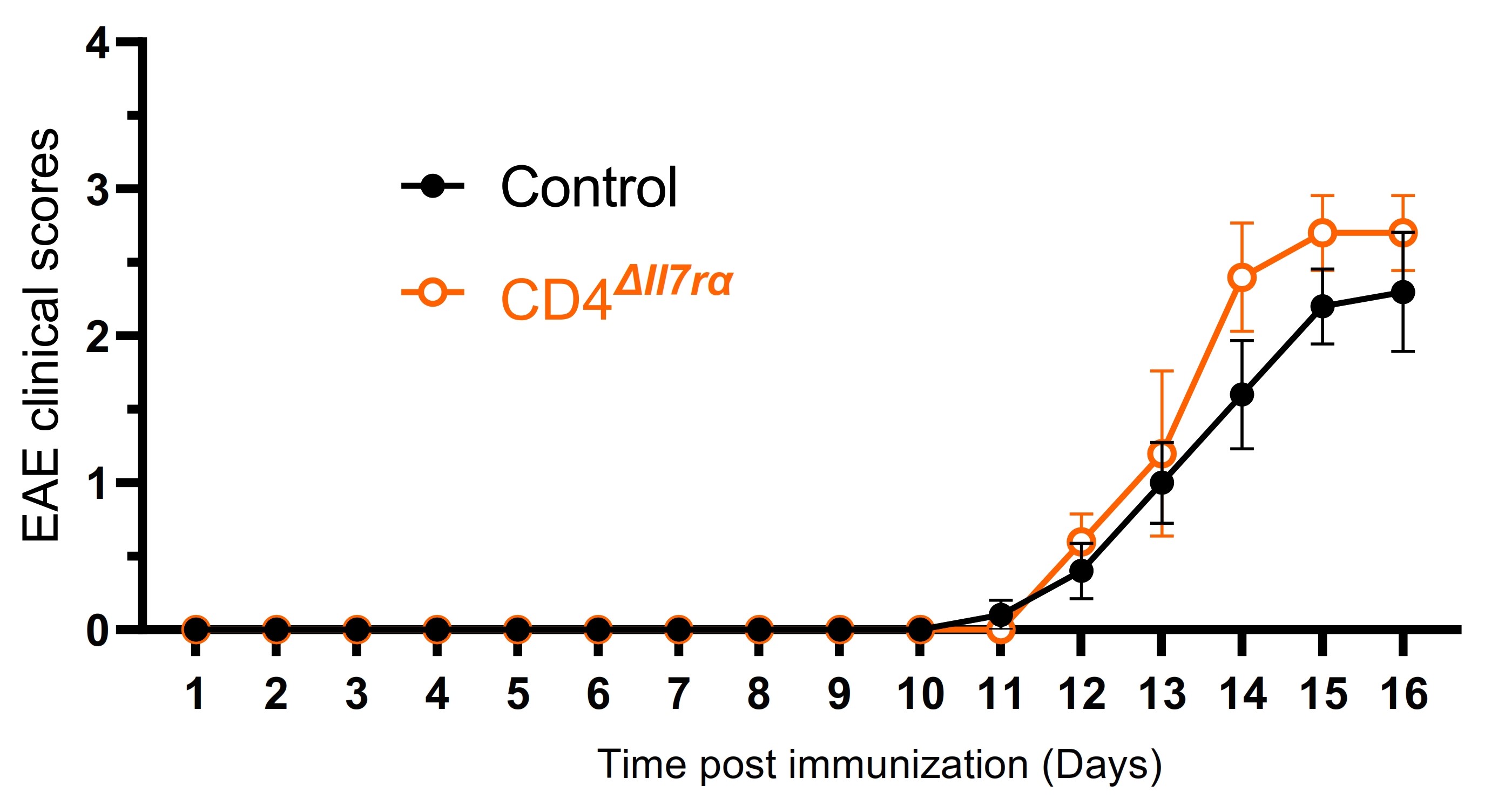

Supplement: Supplementary file 5 — Supplementary Material 5 [file 12974_2024_3224_MOESM5_ESM.jpg]

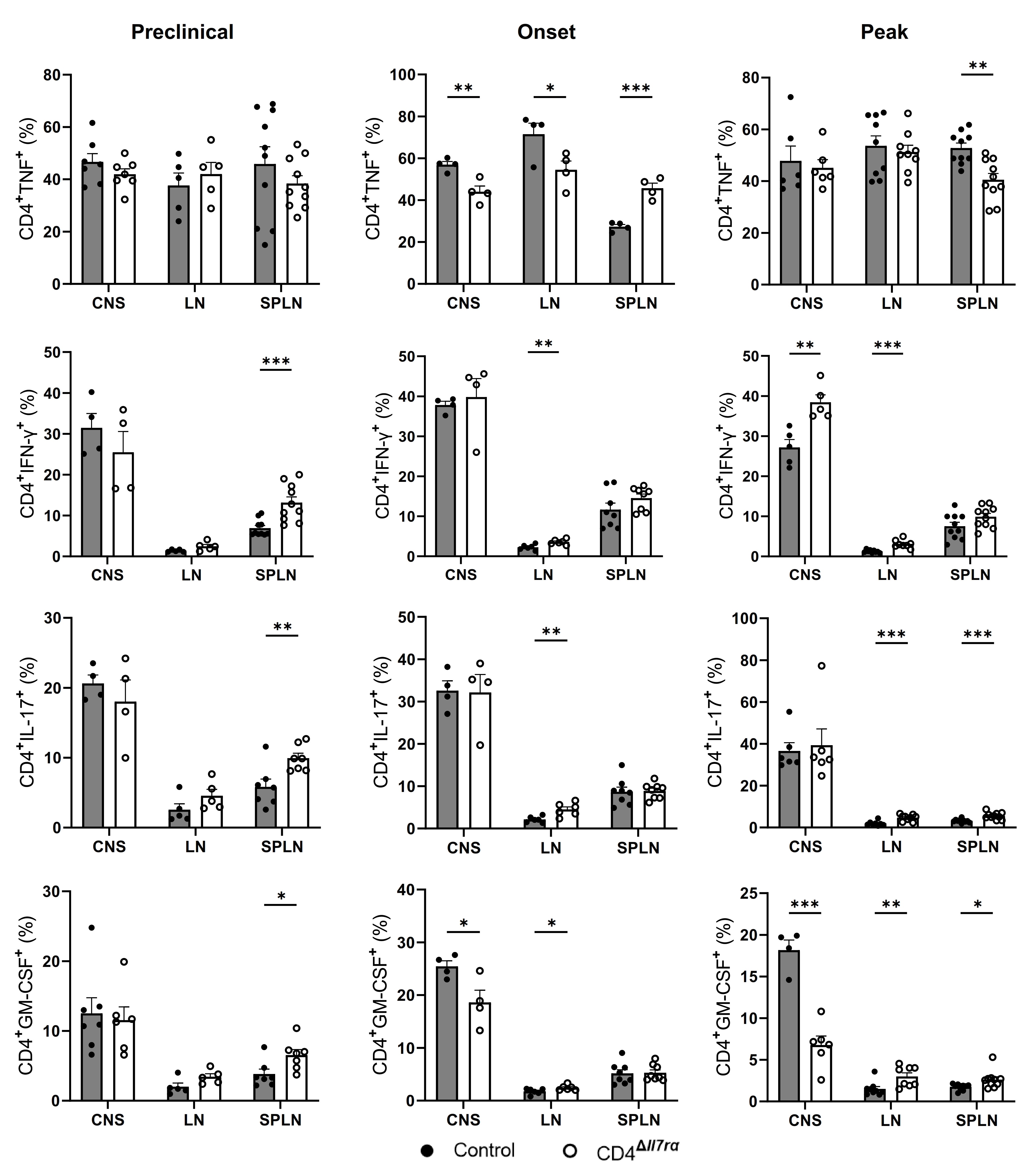

Supplement: Supplementary file 6 — Supplementary Material 6 [file 12974_2024_3224_MOESM6_ESM.jpg]

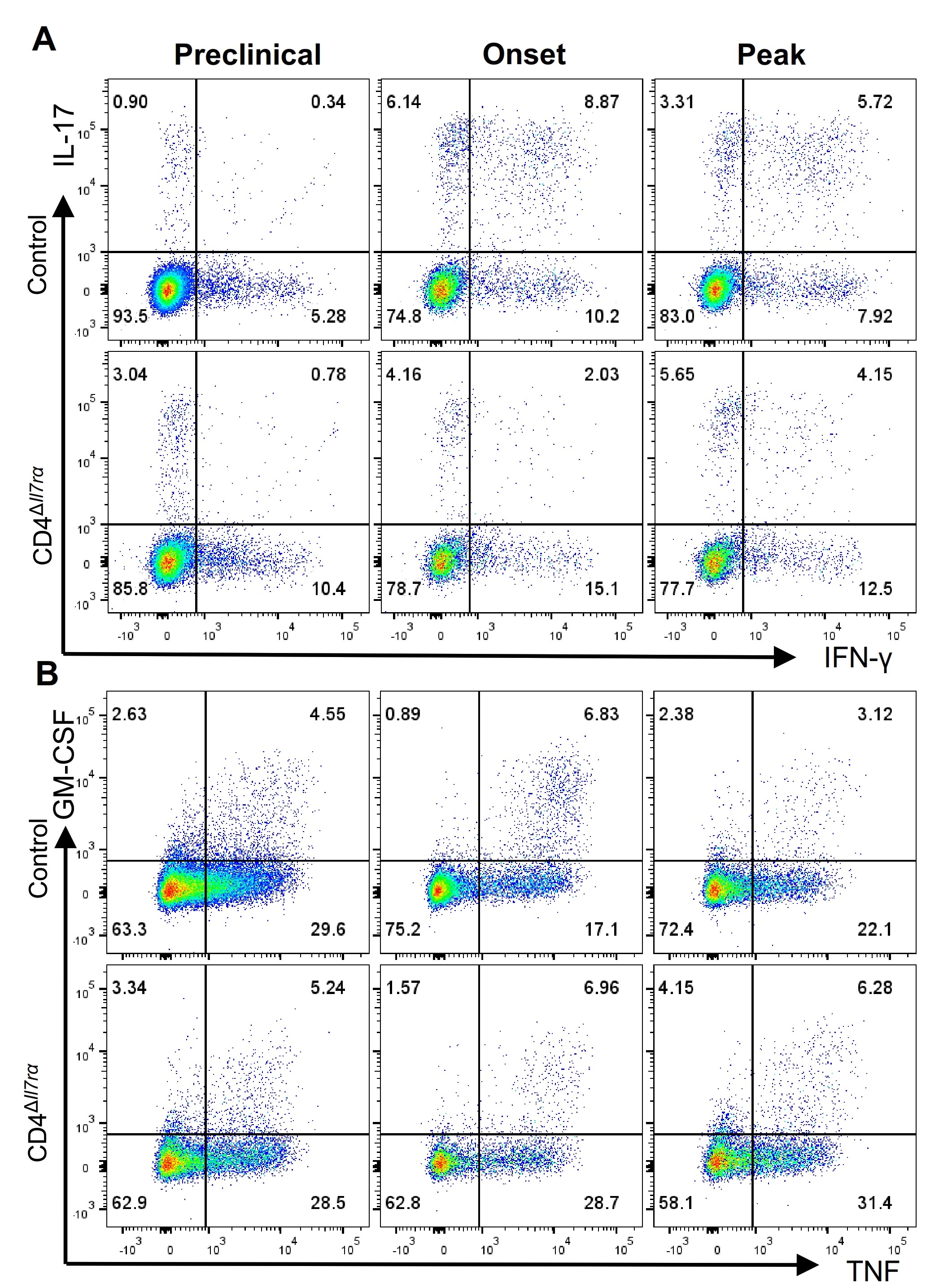

Supplement: Supplementary file 7 — Supplementary Material 7 [file 12974_2024_3224_MOESM7_ESM.jpg]

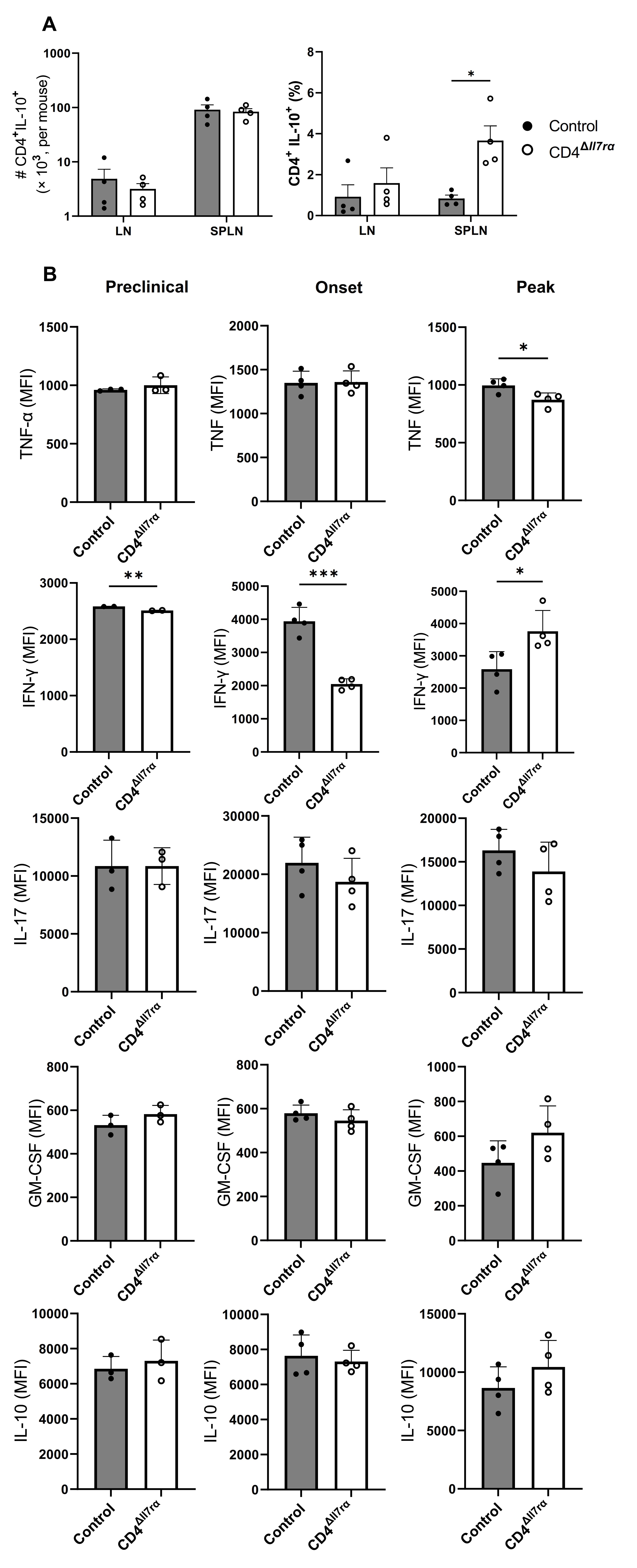

Supplement: Supplementary file 8 — Supplementary Material 8 [file 12974_2024_3224_MOESM8_ESM.jpg]

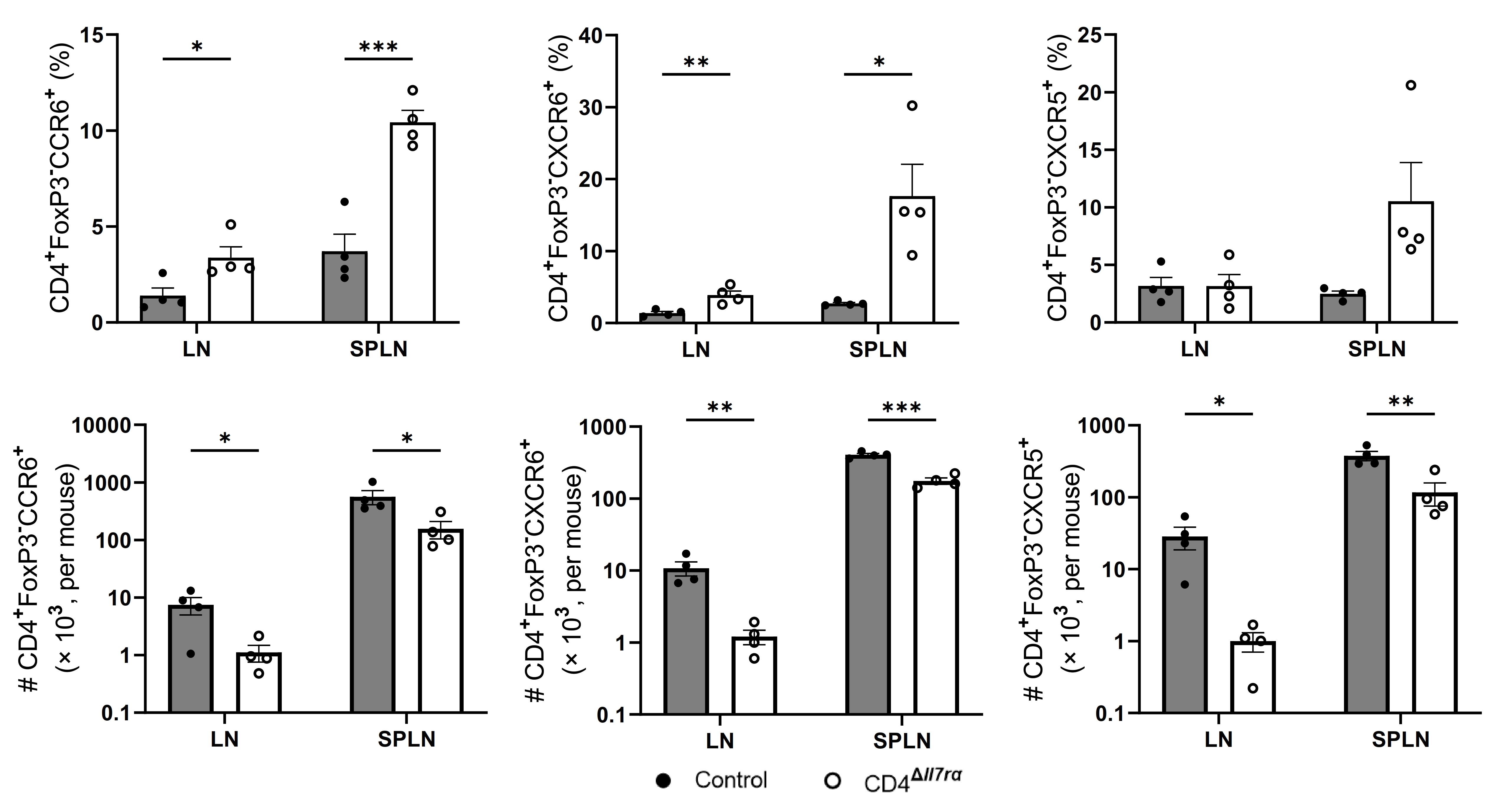

Supplement: Supplementary file 9 — Supplementary Material 9 [file 12974_2024_3224_MOESM9_ESM.jpg]

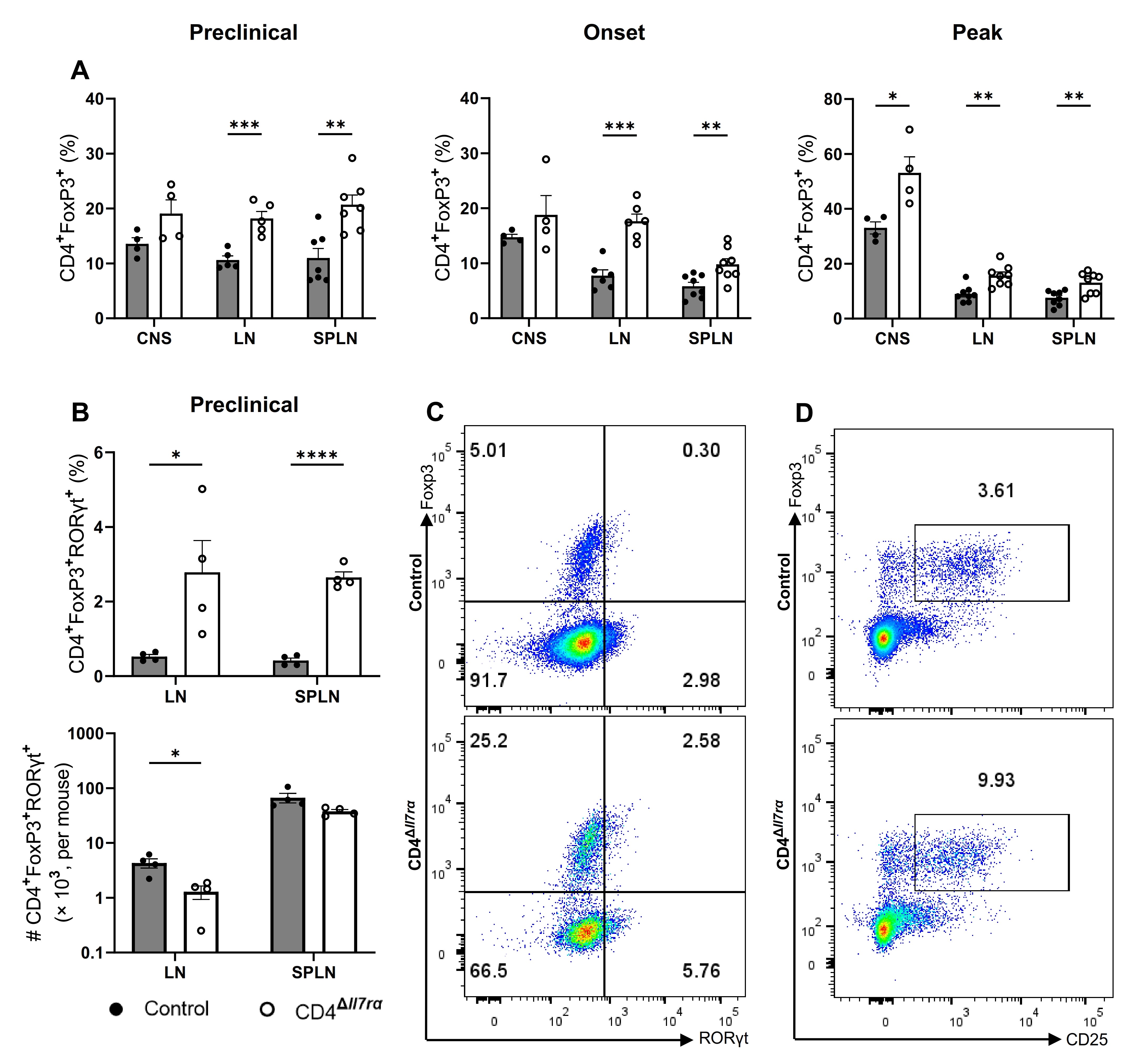

Supplement: Supplementary file 10 — Supplementary Material 10 [file 12974_2024_3224_MOESM10_ESM.jpg]

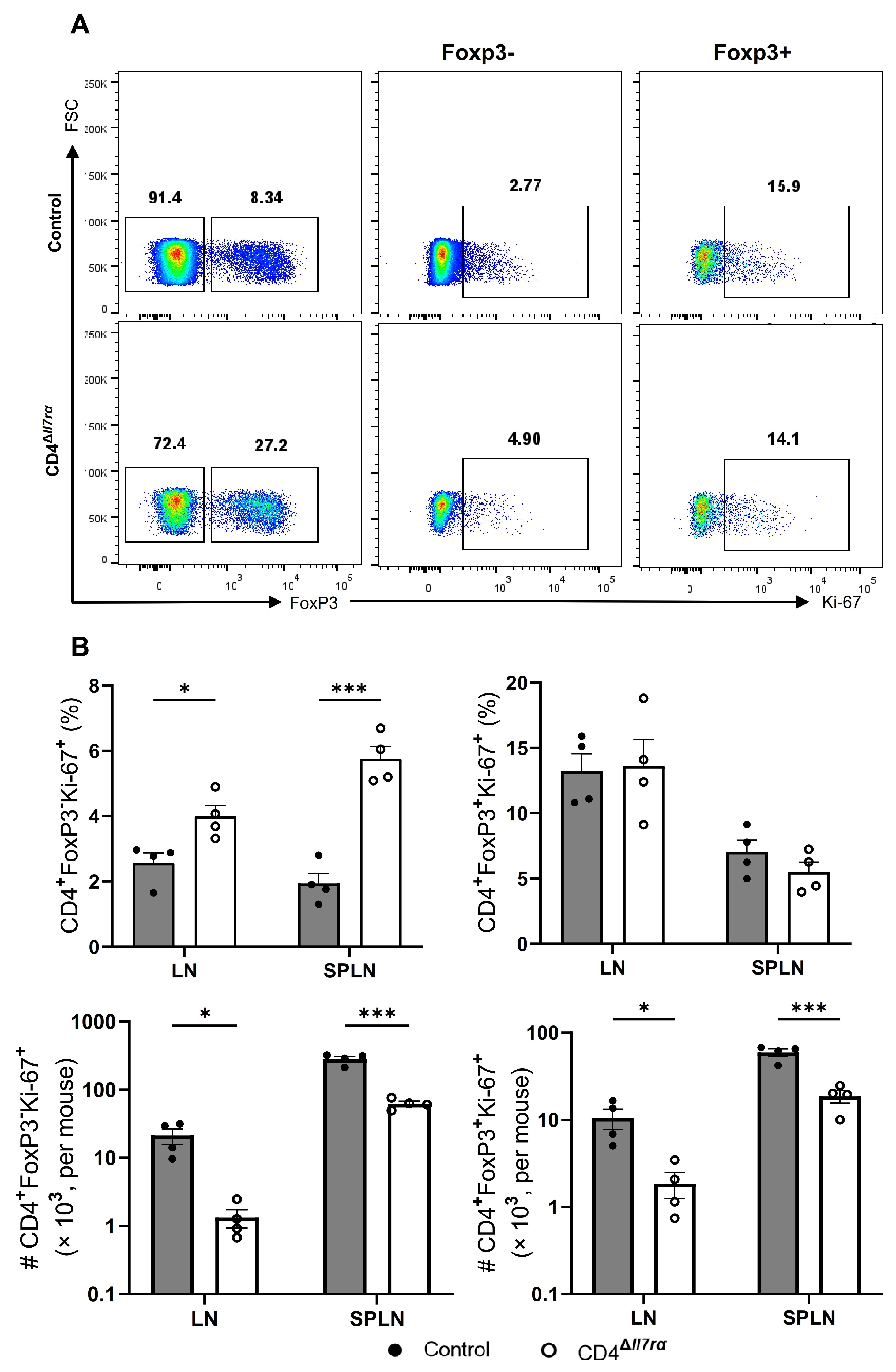

Supplement: Supplementary file 11 — Supplementary Material 11 [file 12974_2024_3224_MOESM11_ESM.jpg]
