# Supplementary material for: IL-7Rα on CD4+ T cells is required for their survival and the pathogenesis of experimental autoimmune encephalomyelitis
Source: J Neuroinflammation. 2024 Oct 8;21:253. doi: 10.1186/s12974-024-03224-2 (PMC11460225; doi:10.1186/s12974-024-03224-2)
Supplement: Supplementary file 12 — Supplementary Material 12 [file 12974_2024_3224_MOESM12_ESM.docx]

**Supplementary material**

**Figure S1. Tamoxifen-induced changes in frequencies of CD4^+^ T cells among mononuclear cells of CD4^Δ^*^Il7ra^* mice.** Three-month-old female CD4^Δ^*^Il7ra^* and control CD4CreER^T2^ mice (n=3-4) were treated once with 250 mg/kg of tamoxifen *via* oral gavage. Mice were sacrificed 144 h after the tamoxifen treatment and evaluated by flow cytometry for IL-7Rα on total, naïve (CD62L^+^CD44^lo^), effector/memory (CD62L^-^CD44^hi^), and regulatory (FoxP3^+^) CD4^+^ T cells and total CD8^+^ T cells from the blood, spleen, LN, and BM. Data are presented as mean ± SEM. The unpaired t-test was used for sample comparisons. *P<0.05; ***P<0.001. The same ratio of Treg cells between the test and control mice was found when Treg cells were defined as CD4^+^CD25^+^Foxp3^+^ or only CD4^+^Foxp3^+^.

**Figure S2. The effect of IL-7Rα** **gene knockout on CD4^+^ T cell frequencies and homeostatic recovery in CD4^Δ^*^Il7ra^* mice.** (**A**) Two-month-old female CD4^Δ^*^Il7ra^* and CD4CreER^T2^ mice were treated with tamoxifen once; and 6 days later, their blood was evaluated for frequencies of naïve and effector/memory cells in gated live total CD4^+^ T cells. (**B**) Two-month-old male and female CD4^Δ^*^Il7ra^* and CD4CreER^T2^ mice were treated with tamoxifen for two consecutive days; and at one, two, and three months, their mononuclear cells from the blood, BM, LNs, and spleen were evaluated for frequencies of total, naïve, effector/memory, and Treg CD4^+^, and total CD8^+^ T cells. Test and control mice sacrificed at each time point were matched for sex and age. Results are expressed as the mean ± SEM with n ≥ 4 per group. The unpaired t-test was used for sample comparisons. *p < 0.05, **p < 0.01, ***p < 0.001, ***p < 0.0001.

**Figure S3. Recovery of IL-7Rα^+^ CD4^+^ T cell population in tamoxifen-treated mice.** (**A**) Two-month-old male and female CD4^Δ^*^Il7ra^* and control CD4CreER^T2^ mice were treated twice with 250 mg/kg of tamoxifen *via* oral gavage for two consecutive days. Mice were sacrificed one, two, and three months later and evaluated by flow cytometry for IL-7Rα on total, naïve, and effector/memory CD4^+^ T cells and total CD8^+^ T cells from the blood, spleen, LN, and BM. (**B**) Mice were treated with two doses of tamoxifen for two consecutive days and then immunized for EAE induction 5 days later. Proportions are shown of IL-7Rα^+^CD4^+^ T cells from blood in CD4^Δ^*^Il7ra^,* and control CD4CreER^T2^ mice with EAE. (**C**) Comparison of EAE courses in tamoxifen pretreated *Il7ra*^fl/fl^ CD4CreER^T2^, WT, and CD4^Δ^*^Il7ra^* mice. Test and control mice sacrificed at each time point were matched for sex and age. Results are expressed as the mean ± SEM with n ≥ 4 (A and B) and n ≥ 2 (C) for each group. Data were analyzed by Student’s t-test and the Kruskal-Wallis test used for clinical score analysis; *P<0.05; **P<0.01; ***P<0.001; ****p<0.0001.

**Figure S4. Reduced frequencies of IL-7Rα^+^ Th cells in CD4^Δ^*^Il7ra^* mice immunized for EAE induction**. Th cells from the CNS, spleen, and LNs of CD4^Δ^*^Il7ra^* and control mice immunized for EAE induction were analyzed for IL-7Rα and cytokine expression at the preclinical phase, onset, and peak of EAE in control mice. Results are expressed as the mean ± SEM with n = 4 per group from 2 independent experiments. Data were analyzed by Student’s t test; *P<0.05; **P<0.01; ***P<0.001; ****P<0.0001.

**Figure S5. CD4^Δ^*^Il7ra^* mice show normal EAE after homeostatic CD4^+^ T cell recovery.** Two-month-old female CD4^Δ^*^Il7ra^* and CD4CreER^T2^ mice were treated with tamoxifen for two consecutive days and rested for 3 months. Mice were then immunized for EAE induction. Clinical scores are shown. Results are expressed as the mean ± SEM with n = 5 per group.

**Figure S6. The frequencies of Th cells in immunized CD4^Δ^*^Il7ra^* mice.** The frequency of cytokine-expressing cells from CD4^Δ^*^Il7ra^* and the control mice during the preclinical phase, onset, and peak of EAE. Results are expressed as the mean ± SEM with n ≥ 4 per group from 2 independent experiments. Data were analyzed by Student’s t-test; *P<0.05; **P<0.01; ***P<0.001.

**Figure S7. Flow cytometry analysis of CD4^+^ T cells from immunized CD4^Δ^*^Il7ra^* and control mice.** Spleen and LN cells were isolated from the CD4^Δ^*^Il7ra^* and control mice at the preclinical phase, EAE onset, and peak. Cells were stimulated with PMA, ionomycin, and GolgiPlug and stained with mAbs against surface and intercellular antigens. Gated CD4^+^ cells are shown. (**A** and **B**) Flow cytometry plots illustrate gating for CD4^+^IFN-γ^+^ and IL-17^+^ cells and CD4^+^TNF^+^ and GM-CSF^+^ cells.

**Figure S8. Cytokine expression by CD4^+^ T cells from mice immunized for EAE induction.** (**A**) The absolute number and frequency of CD4^+^IL-10^+^ cells from CD4^Δ^*^Il7ra^* and the control mice at EAE peak. (**B**) Mean fluorescent intensity (MFI) for TNF, IFN-γ, IL-17, GM-CSF, and IL-10 in CD4^+^ T cells from the spleen of CD4^Δ^*^Il7ra^* and control mice. Results are expressed as the mean ± SEM with n = 4 for each group from 2 independent experiments. Data were analyzed by Student’s t-test; *P<0.05; **P<0.01; ***P<0.001.

**Figure S9. Chemokine receptor expression by CD4^+^ T cells during the preclinical phase of EAE.** Three-month-old male CD4^Δ^*^Il7ra^* and control CD4CreER^T2^ mice were treated twice with 250 mg/kg of tamoxifen *via* oral gavage for two consecutive days. CD4^+^Foxp3^-^ T cells from the spleen and LNs of the mice were analyzed for CCR6, CXCR6, and CXCR5 expression 8 d.p.i. for EAE induction. Results are expressed as the mean ± SEM with n = 4 per group. Data were analyzed by Student’s t-test; *P<0.05; **P<0.01; ***P<0.001.

**Figure S10. The frequencies of Treg cells in immunized CD4^Δ^*^Il7ra^* mice.** (**A**) The frequency of Foxp3^+^CD4^+^ T cells in the CNS, LNs, and spleen of CD4^Δ^*^Il7ra^* mice was compared with control mice during the preclinical phase, onset, and peak of EAE. (**B**) The frequency and absolute number of CD4^+^ Foxp3^+^ RORγt^+^ cells in the spleen of mice 8 d.p.i. for EAE induction. (**C** and **D**) Representative dot plots for CD4^+^ Foxp3^+^ RORγt^+^ and CD4^+^ CD25^+^ Foxp3^+^ cells are shown for the preclinical phase and EAE peak, respectively. Results are expressed as the mean ± SEM, with n ≥ 4 per group from 2 independent experiments, except for the CD4^+^ Foxp3^+^ RORγt^+^ cells, which were analyzed in a single experiment. Data were analyzed by Student’s t-test; *P<0.05; **P<0.01; ***P<0.001; ****P<0.0001.

**Figure S11.** **Ki-67 expression in Tregs and conventional CD4^+^ T cells from mice immunized for EAE induction.** Splenic and LN cells from immunized CD4*^ΔIl7ra^* and control mice were harvested at the preclinical phase of EAE, stained and analyzed by flow cytometry. (**A**) Representative dot plots of CD4^+^Foxp3^-^ and CD4^+^Foxp3^+^ cells and their respective Ki-67^+^ cells from the LNs of CD4*^ΔIl7ra^* and control mice. (**B**) The frequency and absolute number of Ki-67**^+^** cells among CD4**^+^**Foxp3^-^ and CD4^+^Foxp3^+^ cells. Results are expressed as the mean ± SEM, with n = 4 per group. Data were analyzed using Student's t-test; *P<0.05; ***P<0.001.
