# Supplementary material for: IL-7Rα on CD4+ T cells is required for their survival and the pathogenesis of experimental autoimmune encephalomyelitis
Source: J Neuroinflammation. 2024 Oct 8;21:253. doi: 10.1186/s12974-024-03224-2 (PMC11460225; doi:10.1186/s12974-024-03224-2)
Supplement: Supplementary file 13 — Supplementary Material 13 [file 12974_2024_3224_MOESM13_ESM.docx]

**Table S1. Anti-mouse flow cytometry Antibodies.**

| **Antigen** | **Fluorochrome** | **Clone** | **Supplier** | **Category** |
| --- | --- | --- | --- | --- |
| CD4 | FITC | RM4-5 | Biolegend | Surface |
| CD4 | AF700 | GK1.5 | Biolegend | Surface |
| CD8 | PE/Cy7 | 53-6.7 | Biolegend | Surface |
| CD19 | PE/Dazzle594 | 6D5 | Biolegend | Surface |
| CD25 | BV711 | PC61 | Biolegend | Surface |
| CD44 | PE | IM7 | Biolegend | Surface |
| CD62L | APC | MEL-14 | Biolegend | Surface |
| CD127 | PE/Cy5 | A7R34 | Biolegend | Surface |
| CD127 | APC/Cy7 | A7R34 | Biolegend | Surface |
| IL-17 | BV785 | TC11-18H10.1 | Biolegend | Intercellular |
| IL-17 | PE/Dazzle594 | TC11-18H10.1 | Biolegend | Intercellular |
| IFN-γ | BV421 | XMG1.2 | Biolegend | Intercellular |
| IFN-γ | PE/Cy7 | XMG1.2 | Biolegend | Intercellular |
| TNF | FITC | MP6-XT22 | Biolegend | Intercellular |
| TNF | APC/Cy7 | MP6-XT22 | Biolegend | Intercellular |
| GM-CSF | PE | MP1-22E9 | Biolegend | Intercellular |
| GM-CSF | FITC | MP1-22E9 | Biolegend | Intercellular |
| IL-10 | PE | JES5-16E3 | Biolegend | Intercellular |
| IL-10 | PE/Cy7 | JES5-16E3 | Biolegend | Intercellular |
| FoxP3 | eFluor 450 | FJK-16S | eBioscience | Intercellular |
| FoxP3 | PE | FJK-16S | eBioscience | Intercellular |
| RORγt | APC | AFKJS-9 | eBioscience | Intercellular |
| Ki-67 | BV421 | 11F6 | Biolegend | Intercellular |
| CCR6 | PE/Cy7 | 29-2L17 | Biolegend | Surface |
| CXCR6 | PE/Dazzle™ 594 | SA051D1 | Biolegend | Surface |
| CXCR5 | BV785 | L138D7 | Biolegend | Surface |
| IL; Interleukin, IFN; Interferon, TNF; Tumor necrosis factor, GM-CSF; Granulocyte-macrophage colony-stimulating factor, FoxP3; Forkhead box P3, RORγt; retinoic acid-related orphan receptor gamma t, CXCR; CXC chemokine receptor, CCR; C-C chemokine recepor | | | | |
